# Baseline liver fibrosis-4 score correlates to the progression of anxiety and cognitive impairment in patients with Parkinson’s disease

**DOI:** 10.3389/fnagi.2025.1501319

**Published:** 2025-01-24

**Authors:** Yongqing Cheng, Li Chen, Honghong Zhu, Yingchao Ge, Lei Li, Yan Guo, Xin Wang, Shuangfei You, Guojun He, Shouru Xue

**Affiliations:** ^1^Department of Neurology, The Yancheng Clinical College of Xuzhou Medical University, The First People’s Hospital of Yancheng, Yancheng, Jiangsu, China; ^2^Department of Neurology, The First Affiliated Hospital of Soochow University, Suzhou, Jiangsu, China; ^3^Department of Ophthalmology, Funing County People’s Hospital, Yancheng, Jiangsu, China; ^4^Department of Rheumatology and Immunology, The Yancheng Clinical College of Xuzhou Medical University, The First People’s Hospital of Yancheng, Yancheng, Jiangsu, China; ^5^Department of Neurology, Qidong Hospital Affiliated to Nantong University, Nantong, Jiangsu, China; ^6^Department of Radiology, The Yancheng Clinical College of Xuzhou Medical University, The First People’s Hospital of Yancheng, Yancheng, Jiangsu, China

**Keywords:** liver fibrosis-4 score, Parkinson’s disease, progression, cognitive function, anxiety

## Abstract

**Background:**

Non-alcoholic fatty liver disease (NAFLD) or liver fibrosis may share similar pathophysiological features with Parkinson’s disease (PD), yet their correlation was unclear. This study aimed to explore their correlation between PD and liver fibrosis using the fibrosis-4 score (FIB-4) as a surrogate marker.

**Methods:**

We analyzed Parkinson’s Progression Markers Initiative (PPMI) data and enrolled PD patients with comprehensive baseline and 5-year follow-up time-point clinical data. Participants were categorized based on FIB-4 levels to assess the association between FIB-4 scores and various clinical scales, controlling for potential confounders. Differences in the progression of clinical scales over five years were compared using generalized linear mixed models (GLMM).

**Results:**

Baseline FIB-4 levels positively correlated to scores of baseline section III of the Unified-Parkinson Disease Rating Scale (UPDRS III) (*r* = 0.145, *p* = 0.017), Epworth Sleepiness Scale (EPSS) (*r* = 0.140, *P* = 0.022), Hopkins Verbal Learning Test (HVLT)-delayed recall (*r* = 0.128, *P* = 0.036) and HVLT-retention (*r* = 0.128, *p* = 0.036). GLMM analysis revealed an independent correlation between FIB-4 subgroup*time and several clinical scales including the State-trait Anxiety Inventory (STAI), Symbol Digit Modalities Test (SDMT), Semantic Fluency Test (SF), HVLT-total recall, and HVLT-delayed recall, with the high FIB-4 subgroup exhibiting a greater decline in these scores compared to the low FIB-4 subgroup (all *p*<0.05).

**Conclusion:**

Elevated baseline FIB-4 correlated to more severe baseline daytime sleepiness, motor symptoms, and memory function in PD patients, along with a more rapid decline in cognitive functions such as executive function, information processing ability, and memory. Additionally, a high FIB-4 might confer a protective effect against anxiety.

## 1 Introduction

Nonalcoholic fatty liver disease (NAFLD) is the most common chronic liver disease globally, with an estimated incidence of approximately 25% in the general population (Powell et al., 2021). NAFLD a range of pathological conditions from simple steatosis to non-alcoholic steatohepatitis (NASH), with varying degrees of fibrosis and potential progression to cirrhosis (Chalasani et al., 2018). Liver fibrosis has been confirmed to be a predictor the severity, progression, and poor prognosis of NAFLD (Kaya and Yilmaz, 2022; Powell et al., 2021). Beyond its implications for liver diseases, liver fibrosis shares similar risk factors and common pathological mechanisms with cardiovascular diseases and has been shown to be closely associated with the occurrence and poor prognosis of cardiovascular events (Tang et al., 2023; Wen et al., 2022; Liu et al., 2021). The liver-brain axis theory has recently highlighted the potential association between NAFLD/liver fibrosis and neurological disorders (Lombardi et al., 2019; Vegas-Suárez et al., 2022). Although current research is inconclusive, it has been found that NAFLD and liver fibrosis might be associated with brain atrophy, cerebral hypoperfusion, reduced brain activity, cognitive impairment, and stroke severity and prognosis (Lombardi et al., 2019). A few recent studies have reported the potential association between NAFLD and PD but have drawn inconsistent conclusions (Jeong et al., 2021; van Kleef et al., 2023).

The gold standard for liver fibrosis is pathological biopsy, which is is invasive, costly, and carries risks of postoperative complications, making it unsuitable for large-scale screening. Various non-invasive surrogates for assessing liver fibrosis have been proposed based on some physical or biochemical markers (Lai et al., 2024). Fibrosis 4 score (FIB-4), converted from age, aspartate aminotransferase (AST), alanine aminotransferase (ALT), and platelet, is recommended by the American Association for the Study of Liver Diseases (AASLD) as an effective surrogate marker for liver fibrosis (2018). Accumulating evidence has confirmed that FIB-4 could be used to predict NAFLD progression and poor prognosis as well as the occurrence, progression, and poor prognosis of cardiovascular events (Schreiner et al., 2022; Vieira Barbosa et al., 2022a; Anstee et al., 2024). However, the relationship between FIB-4 and Parkinson’s disease has been minimally explored. To the best of our knowledge, there is only one recent research that reported the potential correlation between FIB-4 and cognitive dysfunction in PD (Zolin et al., 2024). Still, it did not study the relationship between FIB-4 and other aspects of PD. Therefore, we designed this study to comprehensively investigate the potential correlation between FIB-4 and PD.

## 2 Materials and methods

Our study comprised both cross-sectional and retrospective cohort analyses, utilizing data from the Parkinson’s Progression Markers Initiative (PPMI).^1^ The PPMI is a extensive, multi-center, longitudinal study designed to collect diverse data to identify biological markers of PD progression (Marek et al., 2011). All data used in this study, details of the study design, procedures, and other protocols are freely accessible online.^2^ The PPMI study was approved by the local Institutional Review Boards of all participating sites, and written informed consent was obtained from each participant. All methods were performed according to the relevant guidelines and regulations. The data was obtained from the PPMI database on November 10, 2023, following the PPMI Data User Agreement rules.

### 2.1 Participants

The inclusion has been reported in detail previously [16]. These criteria required participants to (1) be at least 30 years old at the time of PD diagnosis; (2) exhibit symptoms of asymmetric resting tremor or asymmetric bradykinesia or two symptoms of bradykinesia, resting tremor, and rigidity with a recent diagnosis of PD; (3) have a Hoehn and Yahr (H&Y) stage of 1 or 2; (4) have not received treatment for PD; and (5) have DAT imaging revealing a deficiency in dopamine transporters. Exclusion criteria included patients with atypical PD syndromes, those suspected of having progressive supranuclear palsy, or multiple system atrophy from the follow-up. In addition to meeting the PPMI eligibility criteria, patients who were enrolled in the current study must not have dementia or chronic liver disease at baseline and no missing baseline FIB-4-related hematology data. Meanwhile, eligible participants were required to have complete data on motor and nonmotor assessments at baseline and 5-year follow-up time-point, as well as data from two or more assessments performed annually during the 5-year follow-up.

### 2.2 Clinical characteristics

Demographic characteristics of all subjects were collected, including sex, age, years of education, and disease duration of PD. Section III of the Unified-Parkinson Disease Rating Scale (UPDRS III) and the H&Y stages were used to assess the severity of movement disorders. The common non-motor symptoms were also evaluated. Global non-motor symptoms were assessed using section I of the UPDRS (UPDRS I), and activities of daily living were assessed using section II of the UPDRS (UPDRS II) and the Schwab & England Activities of Daily Living Scale (ADL). Depression was assessed with the Geriatric Depression Scale (GDS), anxiety with the State-Trait Anxiety Inventory (STAI), autonomic nerve function with Scales for Outcomes in Parkinson's disease-Autonomic (SCOPA-AUT), daytime sleepiness with the Epworth Sleepiness Scale (EPSS), REM sleep behavior disorder with the REM Sleep Behavior Disorder Screening Questionnaire (RBDSQ), and impulsive-compulsive behaviors with the Questionnaire for Impulsive-Compulsive Disorders in Parkinson's Disease (QUIP). A comprehensive assessment of cognitive function included global cognition and cognitive subdomains. Montreal Cognitive Assessment (MoCA) was used to assess global cognition, the Benton Judgment of Line Orientation Test (BJLOT) for visuospatial function, the Letter Number Sequencing Test (LNST) for working memory and executive function, the Symbol Digit Modalities Test (SDMT) for attention and speed of information processing and the Semantic Fluency Test (SFT) for semantic memory and executive function, and the Hopkins Verbal Learning Test (HVLT: including total recall, delayed recall, retention and recognition discrimination) for memory function. Olfactory dysfunction, as assessed by the University of Pennsylvania Smell Identification Test (UPSIT), was not included due to a lack of data for follow-up at a 5-year follow-up time-point. The score changes in clinical scales over 5-year follow-up were calculated as the 5-year follow-up time-point scores minus the baseline scores.

### 2.3 Laboratory characteristics

Blood samples were collected, stored, and handled according to the PPMI Research Biomarkers Laboratory manual.^3^ Baseline data collection included pre-enrollment screening or a baseline visit for hematological parameters necessary for FIB-4 calculation, including platelet count, alanine aminotransferase (ALT), and aspartate aminotransferase (AST).

The FIB-4 was calculated using the formula (Sterling et al., 2006):

$\begin{array}{l} \mathrm{FIB}-4=\mathrm{age}\;\left(\mathrm{years}\right)\times AST\;\left(U/L\right)/\\ \;\left[\mathrm{platelet}\;\left(109/L\right)\times \mathrm{ALT}\;{\left(U/L\right)}^{1/2}\right] \end{array}$

Prior research categorizes patients with NAFLD as either low-risk for advanced liver fibrosis with FIB-4 <1.3 or high-risk with FIB-4 ≥ 1.3 (Mcpherson et al., 2017; Zolin et al., 2024). Therefore, Participants were accordingly divided into low FIB-4 and high FIB-4 subgroups based on their baseline FIB-4 levels.

### 2.4 Statistics

Continuous variables are described as mean and standard deviation (SD) or median and interquartile range (IQR) depending on their distribution. Categorical variables were presented as absolute number and percentage (%). The T-test, ANOVA, or Mann-Whitney U test were used to assess differences between the low and high FIB-4 subgroups. Correlations between baseline FIB-4 and clinical scales at baseline and 5-year follow-up time-point were analyzed using Pearman and Spearman correlation analysis, with FIB-4 as a continuous variable. All correlation analyses were adjusted for age, sex, education years, and disease duration of PD. Generalized linear mixed models (GLMM) were then performed to compare the longitudinal changes of clinical variables among patients with different baseline FIB-4 levels (low FIB-4 and high FIB-4 subgroups). The models included the scores of each longitudinal clinical scale, such as UPDRS I, II, III, GDS, etc., as dependent variables. Gender, age, BMI, education years, disease duration, H&Y stages, follow-up time, low FIB-4 subgroup, high FIB-4 subgroup, and the interaction between FIB-4 subgroup and follow-up time (FIB-4 subgroup*time) were used as fixed variables. Then, we conducted a subgroup analysis according to gender and age. Two-sided *P* values < 0.05 were considered to indicate statistical significance. All the statistical analyses and visualization were conducted using SPSS version 23.0 (IBM, New York, NY, USA) and R version 4.2.0.

## 3 Results

### 3.1 Comparison of baseline characteristics between low and high FIB-4 subgroups

A total of 273 PD patients, with a mean age of 60.2 years, were included in the study. The median baseline FIB-4 score was 1.200 (IQR, 0,932, 1.658). Based on the baseline FIB-4 level, participants were divided into a low FIB-4 (*n* = 150) and a high FIB-4 (*n* = 123) subgroups. The baseline characteristics of all subjects, along with comparisons between different subgroups, are detailed in Table 1. In our cohort, patients had a median disease duration of PD of 3.0 (1.0, 6.0) years and a median H&Y stage of 1.0 (1.0, 2.0), with males comprising 66.3% of the study population. Compared to the low FIB-4 subgroup, high FIB-4 subgroup demonstrated a higher proportion of male (60.7% vs. 73.2%, *P* = 0.03) and higher levels of age (mean ± SD, 65.6 ± 7.4 vs. 55.9 ± 9.0, *p* < 0.001), education years (16.00 (13.75, 17.25) vs. 16.0 (15.0, 18.0), *P* = 0.002), H&Y stage (1.0 (1, 2) vs. 2(1, 2), *P* = 0.001), UPDRS III (17 (12, 23) vs. 20 (15, 26), *p* = 0.002) and SCOPA-AUT score (9.5 (6, 16) vs. 12 (8, 18), *P* = 0.019). However, the low FIB-4 subgroup scored higher on cognitive function-related scales, including MoCA (28.00 (26.75, 29.00) vs. 27.00 (26.00, 29.00), *p* = 0.019), LSNT (11 (9, 13) vs. 11 (9, 12), *P* = 0.006) and SDMT (45.5 (39, 50) vs. 39 (34, 46), *p* < 0.001), suggesting better cognitive function in the low FIB-4 subgroup. No significant differences in other baseline characteristics were observed between the two subgroups.

**Table 1:** Comparison of baseline characteristics of PD patients in subgroups with different baseline FIB-4 index levels

Baseline characteristics	Total (*n* = 273)	Low FIB-4 subgroup (*n* = 150)	High FIB-4 subgroup (*n* = 123)	*P* (Low VS higher FIB-4 subgroups)
Age, mean (SD) (year)	60.2 (9.7)	55.9 (9.0)	65.6 (7.4)	<0.001
Male, *n* (%)	181 (66.3)	91 (60.7)	90 (73.2)	0.03
BMI, median (IQR) (Kg/cm^2^)	26.68 (24.06,29.55)	26.99 (24.12,29.63)	26.37 (24.03,29.54)	0.638
Education years, median (IQR) (year)	16 (14,18)	16.00 (13.75,17.25)	16 (15, 18)	0.002
disease duration, median (IQR) (year)	3 (1,6)	3 (1,5)	3 (1,8)	0.136
H&Y stage, median (IQR)	1 (1,2)	1 (1,2)	2 (1,2)	0.001
UPDRS I, median (IQR)	1 (0,2)	1 (0, 2)	1 (0, 1)	0.242
UPDRS II, median (IQR)	4 (2, 7.5)	4 (2, 7)	5 (2,8)	0.152
UPDRS III, median (IQR)	18 (14,24.5)	17 (12,23)	20 (15,26)	0.002
GDS, median (IQR)	5 (4,6)	5 (4,6)	5 (4,6)	0.082
STAI-state, median (IQR)	48 (44,50)	47 (44,50)	49 (45,50)	0.106
STAI-trate, median (IQR)	46 (43.5,48)	46 (43,48)	46 (44,48)	0.398
STAI-total, median (IQR)	93 (88,97.5)	92.5 (87.75,97.0)	94 (90,98)	0.091
QUIP, median (IQR)	4 (4,4)	4 (4,4)	4 (4,4)	0.343
SCOPA-AUT, median (IQR)	10 (6,17)	9.5 (6,16)	12 (8,18)	0.019
EPSS, median (IQR)	5 (3,7.5)	5 (3,7)	6 (3,8)	0.061
RBDSQ, median (IQR)	3 (2,5)	3 (2,5)	3 (2,5)	0.709
ADL, median (IQR)	90 (90,100)	95 (90,100)	90 (90,100)	0.135
MoCA score, median (IQR)	28 (26,29)	28.00 (26.75, 29.00)	27.00 (26.00, 29.00)	0.019
BJOLT, median (IQR)	14 (12,15)	14 (12,15)	14 (12,15)	0.915
LSNT, median (IQR)	11 (9,12)	11 (9,13)	11 (9,12)	0.006
SDMT, median (IQR)	43 (37,49)	45.5 (39,50)	39 (34,46)	<0.001
SFT, median (IQR)	51 (44,57)	50 (43,56)	53 (46,57)	0.123
HVLT Total Recall, mean (SD)	46.83 (10.36)	46.26 (10.04)	47.52 (10.73)	0.318
HVLT Delayed Recall, median (IQR)	47 (38,55)	45 (37,55)	48 (39,55)	0.263
HVLT Retention, median (IQR)	48 (41,55)	48 (41,55)	48 (41,56)	0.504
HVLT Recognition Discrimination, median (IQR)	47 (38,54)	47 (37,57)	47.5 (38,54)	0.373
FIB-4 index, median (IQR)	1.200 (0,932, 1.658)	0.962 (0.789,1.109)	1.678 (1.427,1.966)	<0.001

PD, Parkinson’s Disease; FIB-4, Fibrosis-4; SD, standard deviation; BMI, body mass index; IQR, range interquartile; MDS-UPDRS I, II, III, Part I, II, III of Unified Parkinson's Disease Rating Scale; GDS, Geriatric Depression Scale; STAI, State–Trait Anxiety Inventory; QUIP, Questionnaire for Impulsive-Compulsive Disorders in Parkinson's Disease (QUIP); SCOPA-AUT, Scales for Outcomes in Parkinson's disease-Autonomic; EPSS, Epworth Sleepiness Scale; RBDSQ, REM sleep behavior disorder with the REM Sleep Behavior Disorder Screening Questionnaire; ADL, Schwab & England Activities of Daily Living Scale; MoCA, Montreal Cognitive Assessment; BJOLT, Benton Judgment of Line Orientation Test; LNST, Letter Number Sequencing Test; SDMT, Symbol Digit Modalities Test; SFT, Semantic Fluency Test; HVLT, Hopkins Verbal Learning Test.

### 3.2 Comparison of score changes in clinical scales between low and high FIB-4 subgroups over 5-year follow-up

Table 2 presents the differences in the score changes of the clinical scales between baseline and the 5-year follow-up time-point (calculated as the 5-year follow-up time-point score minus the baseline score). Significant differences in the changes related to anxiety and cognition were observed between the subgroups. After 5 years of follow-up, the STAI scores (including state, trait, and total scores) of patients in the high FIB-4 subgroup decreased more substantially than those in the low FIB-4 subgroup (*P* = 0.005 for state, *P* = 0.023 for trait, and *P* = 0.002 for total). In addition, compared to the low FIB-4 subgroup, the high FIB-4 subgroup showed greater decreases in cognitive-related scales, including LSNT, SDMT, SFT, HVLT total recall, and HVLT recognition discrimination (*P* = 0.012, *P* = 0.011, *P* = 0.022, *P* = 0.015, and *p* = 0.023, respectively). However, no significant differences were observed in the global cognitive-related MoCA, motor-related UPDRS III, or other clinical scales between the two subgroups.

**Table 2:** Comparison of score changes in clinical scales between low FIB-4 and high FIB-4 subgroups over 5-year follow-up

Clinical scale	Total	Low FIB-4 subgroup	High FIB-4 subgroup	*P* (Low VS higher FIB-4 subgroups)
UPDRS I, median (IQR)	0 (0,2)	0 (−1,2)	0 (0,2)	0.156
UPDRS II, median (IQR)	4 (0,8)	4 (0,7.25)	3 (0,8)	0.841
UPDRS III, median (IQR)	8 (2.0,17.5)	8 (3,18)	8 (0,17)	0.256
GDS, median (IQR)	0 (−1,1)	0 (−1,1)	0 (−1,1)	0.482
STAI-state, median (IQR)	0 (−4.75,3.0)	0 (−4,4)	−1 (−5,2)	0.005
STAI-trait, median (IQR)	−1 (−3,2.0)	−1 (−3,3)	−1 (−4,2)	0.023
STAI-total, median (IQR)	−1 (−6,4)	1 (−5,5)	−1 (−7,2)	0.002
QUIP, median (IQR)	−4 (−4,−1)	−4 (−4,−1)	−4 (−4,−1)	0.447
SCOPA, median (IQR)	5 (0,9.5)	5 (0,9)	5 (0,10)	0.593
EPSS, median (IQR)	2 (−1,5)	2 (0,5)	1 (−1,4)	0.322
RBDSQ, median (IQR)	0 (−1,2)	0 (−1,2.25)	1 (−1,2)	0.541
ADL, median (IQR)	−10 (−10,0)	−10 (−10,0)	−10 (−10,0)	0.641
MoCA score, median (IQR)	0 (−2,1)	0 (−1.25,1)	0 (−2,1)	0.115
BJOLT, median (IQR)	0 (−2,1)	0 (−1,1)	−1 (−2,1)	0.111
LSNT, median (IQR)	0 (−2,1)	0 (−2,1)	−1 (−2,0)	0.012
SDMT, median (IQR)	−2 (−7,5)	−1 (−6,6)	−2 (−8,3)	0.011
SFT, median (IQR)	1 (−6,8.5)	2 (−6,9)	−2 (−6,6)	0.022
HVLT Total Recall, mean (SD)	1.69 (11.84)	3.43 (11.62)	−0.44 (11.80)	0.015
HVLT Delayed Recall, median (IQR)	2 (−3,11)	4.5 (−3,12)	2 (−4,10)	0.083
HVLT Retention, median (IQR)	2 (−6,11)	2 (−5,12)	2 (−8,10)	0.510
HVLT Recognition Discrimination, median (IQR)	0.5 (−4,11)	4 (−3,11.25)	−1.5 (−8,9)	0.023

FIB-4, Fibrosis-4; SD, standard deviation; IQR, range interquartile; UPDRS I, II, III, Part I, II, III of Unified Parkinson's Disease Rating Scale; GDS, Geriatric Depression Scale; STAI, State–Trait Anxiety Inventory; QUIP, Questionnaire for Impulsive-Compulsive Disorders in Parkinson's Disease (QUIP); SCOPA-AUT, Scales for Outcomes in Parkinson's disease-Autonomic; EPSS, Epworth Sleepiness Scale; RBDSQ, REM sleep behavior disorder with the REM Sleep Behavior Disorder Screening Questionnaire; ADL, Schwab & England Activities of Daily Living Scale; MoCA, Montreal Cognitive Assessment; BJOLT, Benton Judgment of Line Orientation Test; LNST, Letter Number Sequencing Test; SDMT, Symbol Digit Modalities Test; SFT, Semantic Fluency Test; HVLT, Hopkins Verbal Learning Test.

### 3.3 Correlation analysis between baseline FIB-4 index and clinical scale scores at baseline and 5-year follow-up time-point

The correlation analysis results between baseline FIB-4 level and clinical scales are shown in Table 3. Baseline FIB-4 levels were positively correlated with baseline UPDRS III (*r* = 0.145, *p* = 0.017), EPSS (*r* = 0.140, *p* = 0.022), HVLT delayed recall (*r* = 0.128, *P* = 0.036) and HVLT retention scores (*r* = 0.128, *p* = 0.036). At the 5-year follow-up, baseline FIB-4 was still positively correlated with EPSS but not with other clinical scales.

**Table 3:** Correlation analysis between baseline FIB-4 index and clinical scale scores at baseline and 5-year follow-up time point

Clinical scales	Baseline		5 year follow-up time-point	
	*r*	*p*	*r*	*p*
UPDRS I	−0.028	0.649	0.014	0.825
UPDRS II	0.085	0.166	0.005	0.939
UPDRS III	0.145	0.017	0.013	0.832
GDS	0.014	0.815	0.012	0.840
STAI-state	0.061	0.322	−0.021	0.728
STAI-trait	0.082	0.180	0.068	0.310
STAI-total	0.083	0.174	0.019	0.761
QUIP	0.013	0.827	−0.006	0.919
SCOPA-AUT	0.010	0.866	0.115	0.060
EPSS	0.140	0.022	0.146	0.016
RBDSQ	0.043	0.479	0.044	0.471
ADL	−0.023	0.711	−0.054	0.381
MoCA score	0.017	0.783	−0.034	0.581
BJOLT	0.017	0.779	−0.116	0.058
LSNT	−0.029	0.638	−0.096	0.117
SDMT	−0.046	0.451	−0.072	0.237
SFT	0.027	0.661	0.040	0.517
HVLT Total Recall	0.048	0.431	−0.077	0.210
HVLT Delayed Recall	0.128	0.036	0.010	0.870
HVLT Retention	0.128	0.036	0.040	0.509
HVLT Recognition Discrimination	0.090	0.143	−0.074	0.229

FIB-4, Fibrosis-4; UPDRS I, II, III, Part I, II, III of Unified Parkinson's Disease Rating Scale; GDS, Geriatric Depression Scale; STAI, State–Trait Anxiety Inventory; QUIP, Questionnaire for Impulsive-Compulsive Disorders in Parkinson's Disease (QUIP); SCOPA-AUT, Scales for Outcomes in Parkinson's disease-Autonomic; EPSS, Epworth Sleepiness Scale; RBDSQ, REM sleep behavior disorder with the REM Sleep Behavior Disorder Screening Questionnaire; ADL, Schwab & England Activities of Daily Living Scale; MoCA, Montreal Cognitive Assessment; BJOLT, Benton Judgment of Line Orientation Test; LNST, Letter Number Sequencing Test; SDMT, Symbol Digit Modalities Test; SFT, Semantic Fluency Test; HVLT, Hopkins Verbal Learning Test.

### 3.4 The prediction of longitudinal changes in clinical scales using baseline FIB-4

**Figure 1:**
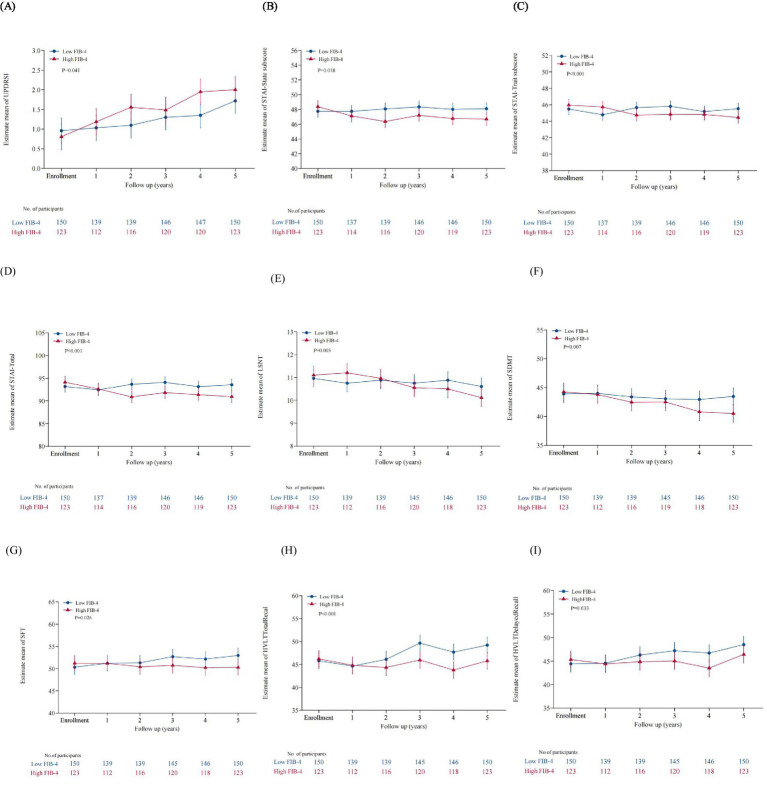
Comparison of clinical scales with significant differences in longitudinal change trends between the two subgroup. **(A)** The UPDRS I scores of the high FIB-4 subgroup showed a faster longitudinal increase over the five-year period. **(B)** The STAI-State subscore of the high FIB-4 subgroup showed a faster longitudinal decline. **(C)** The STAI-Trate subscore of the high FIB-4 subgroup showed a faster longitudinal decline. **(D)** Showed a faster longitudinal decline. **(E)** The LSNT of the high FIB-4 subgroup showed a faster longitudinal decline. **(F)** The SDMT of the high FIB-4 subgroup showed a fasterlongitudinal decline. **(G)** The SFT of the high FIB-4 subgroup showed a faster longitudinal decline. **(H)** The HVLT Total Recal of the high FIB-4 subgroup showed a faster longitudinal decline. **(I)** The HVLT Delayed Recal of the high FIB-4 subgroup showed a faster longitudinal decline.

To elucidate the relationship between baseline FIB-4 levels and the progression of PD further, we examined longitudinal changes in clinical scales in patients with low and high levels of baseline FIB-4. General linear mixed-effect models (GLMM) were conducted to compare the progression of clinical symptoms between groups by testing the interaction between subgroups and time (FIB-4 subgroup*time). The results showed that FIB-4 subgroup*time was independently correlated with UPDRS I, STAI, LSNT, SDMT, SFT, HVLT-total recall, and HVLT-delayed recall scores (Table 4; Figure 1). In particular, patients in the high FIB-4 subgroup showed a significantly faster decline in cognitive subdomain scores at late follow-up, including LSNT, SDMT, SF, HVLT total recal and HVLT delayed recall. This suggests that high levels of FIB-4 may aggravate the deterioration of cognitive function with the prolongation of disease duration. Meanwhile, the anxiety-related scale scores, including the STAI-state subscore, the STAI-trait subscore, and the STAI-total scores, decreased more significantly in the high FIB-4 level subgroup over the 5-year period. It is suggested that high level of FIB-4 may have a potential protective effect on anxiety in PD patients. There were no significant differences in the longitudinal changes of other clinical scales scores during the 5-year follow-up period (Supplementary Table S1).

**Table 4:** Comparison of the longitudinal change trend of cognitive and anxiety related scale scores in different FIB-4 subgroups over 5 years using general linear mixed-effect models.

Clinical scales	Low FIB-4 subgroup	High FIB-4 subgroup	Difference in Low FIB-4 subgroup (95% CI)	Difference in high FIB-4 subgroup (95% CI)	Difference between subgroups (95% CI)	P^a^	P^b^
UPDRSI, estimated mean (SD)							0.041
Baseline	0.96 (0.32)	0.81 (0.33)	-	-	−0.15 (−0.59, 0.28)	0.492	
1	1.03 (0.32)	1.18 (0.33)	0.07 (−0.25, 0.40)	0.38 (0.02, 0.74)	0.30 (−0.18, 0.79)	0.216	
2	1.10 (0.32)	1.56 (0.33)	0.14 (−0.18, 0.46)	0.75 (0.39, 1.10)	0.61 (0.13, 1.09)	0.013	
3	1.30 (0.32)	1.48 (0.33)	0.34 (0.02, 0.66)	0.68 (0.33, 1.03)	0.34 (−0.14, 0.81)	0.162	
4	1.35 (0.32)	1.95 (0.33)	0.39 (0.07, 0.71)	1.14 (0.79, 1.49)	0.75 (0.28, 1.22)	0.002	
5	1.72 (0.32)	2.00 (0.33)	0.76 (0.44, 1.08)	1.20 (0.85, 1.54)	0.44 (−0.04, 0.91)	0.070	
STAI-State subscore, estimated mean (SD)							0.018
Baseline	47.75 (0.78)	48.37 (0.82)	-	-	0.62 (−0.54, 1.79)	0.294	
1	47.73 (0.79)	47.12 (0.82)	−0.02 (−0.98, 0.94)	−1.25 (−2.31, −0.20)	−1.23 (−2.66, 0.19)	0.089	
2	48.07 (0.79)	46.37 (0.82)	0.32 (−0.63, 1.28)	−2.00 (−3.05, −0.96)	−2.33 (−3.74, −0.91)	0.001	
3	48.34 (0.79)	47.21 (0.82)	0.59 (−0.35, 1.53)	−1.17 (−2.21, −0.13)	−1.76 (−3.16, −0.36)	0.014	
4	48.02 (0.79)	46.77 (0.82)	0.27 (−0.67, 1.21)	−1.60 (−2.64, −0.56)	−1.87 (−3.27, −0.47)	0.009	
5	48.10 (0.78)	46.70 (0.82)	0.35 (−0.59, 1.28)	−1.67 (−2.70, −0.64)	−2.02 (−3.41, −0.63)	0.004	
STAI-Trait subscore, estimated mean (SD)							<0.001
Baseline	45.48 (0.66)	45.96 (0.68)	-	-	0.48 (−0.46, 1.42)	0.314	
1	44.78 (0.66)	45.73 (0.69)	−0.70 (−1.44, 0.04)	−0.24 (−1.05, 0.57)	0.46 (−0.63, 1.56)	0.407	
2	45.65 (0.66)	44.74 (0.69)	0.17 (−0.56, 0.91)	−1.22 (−2.03, −0.41)	−1.39 (−2.48, −0.30)	0.013	
3	45.80 (0.66)	44.83 (0.69)	0.32 (−0.40, 1.05)	−1.13 (−1.93, −0.33)	−1.45 (−2.53, −0.37)	0.008	
4	45.18 (0.66)	44.82 (0.69)	−0.31 (−1.03, 0.42)	−1.14 (−1.95, −0.34)	−0.84 (−1.92, 0.24)	0.129	
5	45.52 (0.66)	44.46 (0.68)	0.04 (−0.68, 0.76)	−1.50 (−2.30, −0.71)	−1.54 (−2.62, −0.47)	0.005	
STAI-Total, estimated mean (SD)							<0.001
Baseline	93.16 (1.22)	94.11 (1.27)	-	-	0.96 (−0.79, 2.70)	0.283	
1	92.43 (1.23)	92.61 (1.28)	−0.73 (−2.11, 0.65)	−1.50 (−3.02, 0.02)	−0.77 (−2.82, 1.28)	0.461	
2	93.65 (1.23)	90.88 (1.27)	0.49 (−0.88, 1.87)	−3.23 (−4.74, −1.72)	−3.72 (−5.76, −1.68)	<0.001	
3	94.07 (1.22)	91.82 (1.27)	0.92 (−0.44, 2.27)	−2.29 (−3.79, −0.80)	−3.21 (−5.23, −1.19)	0.002	
4	93.12 (1.22)	91.36 (1.27)	−0.04 (−1.39, 1.32)	−2.75 (−4.25, −1.25)	−2.71 (−4.73, −0.69)	0.009	
5	93.54 (1.22)	90.93 (1.27)	0.39 (−0.96, 1.73)	−3.18 (−4.66, −1.69)	−3.57 (−5.57, −1.56)	<0.001	
LSNT, estimated mean (SD)							0.005
Baseline	10.96 (0.37)	11.10 (0.39)	-	-	0.14 (−0.36, 0.64)	0.579	
1	10.75 (0.38)	11.21 (0.39)	−0.21 (−0.57, 0.15)	0.11 (−0.30, 0.51)	0.32 (−0.23, 0.86)	0.252	
2	10.88 (0.38)	10.96 (0.39)	−0.08 (−0.44, 0.29)	−0.14 (−0.54, 0.26)	−0.06 (−0.60, 0.48)	0.821	
3	10.75 (0.38)	10.55 (0.39)	−0.21 (−0.57, 0.15)	−0.55 (−0.94, −0.15)	−0.34 (−0.87, 0.19)	0.211	
4	10.88 (0.37)	10.50 (0.39)	−0.08 (−0.44, 0.28)	−0.60 (−1.00, −0.21)	−0.52 (−1.06, 0.01)	0.054	
5	10.61 (0.37)	10.12 (0.39)	−0.35 (−0.71, 0.00)	−0.98 (−1.38, −0.59)	−0.63 (−1.16, −0.10)	0.019	
SDMT, estimated mean (SD)							0.007
Baseline	43.89 (1.46)	44.25 (1.51)	-	-	0.36 (−1.53, 2.25)	0.711	
1	43.99 (1.47)	43.78 (1.52)	0.10 (−1.22, 1.41)	−0.47 (−1.93, 0.99)	−0.57 (−2.53, 1.40)	0.574	
2	43.38 (1.47)	42.45 (1.51)	−0.51 (−1.83, 0.81)	−1.80 (−3.25, −0.35)	−1.29 (−3.24, 0.67)	0.198	
3	43.05 (1.46)	42.49 (1.51)	−0.84 (−2.14, 0.46)	−1.76 (−3.19, −0.32)	−0.92 (−2.86, 1.02)	0.353	
4	42.93 (1.46)	40.80 (1.51)	−0.96 (−2.26, 0.34)	−3.45 (−4.89, −2.01)	−2.49 (−4.43, −0.55)	0.012	
5	43.46 (1.46)	40.51 (1.51)	−0.43 (−1.72, 0.86)	−3.74 (−5.16, −2.32)	−3.31 (−5.23, −1.39)	0.001	
SFT, estimated mean (SD)							0.026
Baseline	50.32 (1.64)	51.22 (1.70)	-	-	0.90 (−1.29, 3.08)	0.421	
1	51.21 (1.65)	51.19 (1.71)	0.88 (−0.71, 2.48)	−0.03 (−1.81, 1.74)	−0.92 (−3.30, 1.47)	0.452	
2	51.31 (1.65)	50.41 (1.70)	0.98 (−0.61, 2.58)	−0.81 (−2.57, 0.94)	−1.80 (−4.17, 0.58)	0.138	
3	52.69 (1.64)	50.73 (1.70)	2.37 (0.79, 3.95)	−0.50 (−2.23, 1.24)	−2.87 (−5.21, −0.52)	0.017	
4	52.15 (1.64)	50.22 (1.70)	1.83 (0.25, 3.40)	−1.00 (−2.75, 0.74)	−2.83 (−5.18, −0.48)	0.018	
5	52.97 (1.64)	50.29 (1.70)	2.65 (1.08, 4.21)	−0.93 (−2.65, 0.80)	−3.57 (−5.90, −1.25)	0.003	
HVLT total recall, estimated mean (SD)							<0.001
Baseline	45.79 (1.72)	46.23 (1.78)	-	-	0.44 (−1.87, 2.74)	0.710	
1	44.64 (1.73)	44.82 (1.79)	−1.15 (−2.83, 0.53)	−1.41 (−3.28, 0.46)	−0.26 (−2.77, 2.25)	0.840	
2	46.12 (1.73)	44.34 (1.79)	0.33 (−1.35, 2.02)	−1.89 (−3.74, −0.04)	−2.22 (−4.72, 0.28)	0.081	
3	49.66 (1.73)	45.97 (1.78)	3.87 (2.20, 5.53)	−0.26 (−2.09, 1.57)	−4.12 (−6.59, −1.65)	0.001	
4	47.70 (1.73)	43.77 (1.79)	1.91 (0.25, 3.57)	−2.46 (−4.30, −0.62)	−4.36 (−6.84, −1.89)	0.001	
5	49.22 (1.72)	45.79 (1.78)	3.43 (1.79, 5.08)	−0.44 (−2.26, 1.38)	−3.87 (−6.32, −1.42)	0.002	
HVLT delayed recall, estimated mean (SD)							0.033
Baseline	44.42 (1.75)	45.33 (1.81)	-	-	0.91 (−1.48, 3.30)	0.455	
1	44.55 (1.75)	44.40 (1.82)	0.13 (−1.67, 1.94)	−0.93 (−2.93, 1.07)	−1.06 (−3.75, 1.64)	0.441	
2	46.29 (1.75)	44.87 (1.82)	1.87 (0.07, 3.67)	−0.46 (−2.44, 1.52)	−2.33 (−5.01, 0.35)	0.088	
3	47.21 (1.75)	45.02 (1.81)	2.79 (1.01, 4.57)	−0.31 (−2.27, 1.65)	−3.10 (−5.75, −0.45)	0.022	
4	46.72 (1.75)	43.52 (1.81)	2.30 (0.52, 4.08)	−1.81 (−3.78, 0.17)	−4.11 (−6.76, −1.45)	0.002	
5	48.50 (1.75)	46.42 (1.81)	4.09 (2.32, 5.85)	1.10 (−0.85, 3.05)	−2.99 (−5.62, −0.36)	0.026	

FIB-4, Fibrosis-4; UPDRS I, II, III, Part I, II, III of Unified Parkinson's Disease Rating Scale; STAI, State–Trait Anxiety Inventory; LNST, Letter Number Sequencing Test; SDMT, Symbol Digit Modalities Test; SFT, Semantic Fluency Test; HVLT, Hopkins Verbal Learning Test. P^a^: P for Difference between subgroups; P^b^: P for FIB-4 subgroup*time.

### 3.5 Subgroup analyses based on age and sex

In order to determine whether the association between baseline FIB-4 levels and progression of cognitive impairment was related to age and gender, subgroup analyses were performed according to age and gender, respectively. The results showed that the differences in longitudinal cognitive changes between different FIB-4 levels subgroups persisted in male PD patients, but not in female patients (Supplementary Tables S2, S3). Meanwhile, in PD patients older than 60 years old, the progression of cognitive impairment was also significantly different between the two high and low FIB-4 subgroups, but there was no significant difference in PD patients younger than 60 years old (Supplementary Tables S4, S5).

## 4 Discussion

In this study, we explored the association of baseline FIB-4 levels with clinical manifestations of PD at baseline and after 5-year follow-up, through both cross-sectional and cohort analyses. Our study discovered that patients with elevated FIB-4 levels exhibited more pronounced baseline motor and autonomic dysfunctions, as well as cognitive impairments. The baseline FIB-4 level significantly correlated with the baseline UPDRS III score, EPSS, HVLT delayed recall, and HVLT retention, even after adjusting for age, sex, disease duration, and education level. The results affirmed that a higher baseline FIB-4 was related to severe baseline PD motor deficits, daytime sleepiness, and cognitive impairment. However, at the 5-year follow-up time-point, the baseline FIB-4 levels were only correlated with the EPSS, but not with other clinical characteristics, hinting that baseline FIB-4 might predict the severity of daytime sleepiness in PD patients after five years. Moreover, our cohort analysis revealed a link between baseline FIB-4 levels and the evolution of PD-related anxiety and cognitive changes over time. Notably, there were significant differences in the changes observed in the STAI, LSNT, SDMT, SFT, HVLT total recall, and HVLT recognition discrimination scores between patients with high and low FIB-4 levels. The results of the GLMM analysis confirmed that patients with elevated FIB-4 levels experienced faster longitudinal progression in SDMT, SFT, HVLT-total recall, HVLT recognition discrimination, and a slower longitudinal progression in STAI scores. Consistent with prior research, our study also illustrated that a high baseline level of FIB-4 accelerated the deterioration of cognitive functions, including executive function, information processing ability, and memory, albeit not affecting global cognition (Zolin et al., 2024). For the first time, our findings suggest that high baseline FIB-4 levels might offer protective benefits against anxiety in PD. In addition, previous studies have shown that the sensitivity and specificity of FIB-4 in the evaluation of liver fibrosis are different in different gender and age groups. Similarly, our study also suggested that the effect of FIB-4 on the longitudinal changes of cognitive function was different in different gender and age groups. However, since this was a *post hoc* analysis, the differences in other indicators between the subgroups could not be controlled, and the sample sizes of the subgroups were not balanced, so further studies are needed to confirm this conclusion.

The role of liver fibrosis scores, such as FIB-4, has primarily been associated with assessing the severity and prognosis of liver diseases, as they considerably predict liver disease risk and adverse outcomes (Albhaisi et al., 2023). A prospective cohort study in the United States indicated that higher liver fibrosis index scores correlate with increased liver disease incidence and overall mortality, even in individuals without viral hepatitis (Unalp Arida and Ruhl, 2017). Another study conducted by Cholankeril et al. showed that longitudinal changes of FIB-4 were strongly associated with progression to cirrhosis and hepatocellular carcinoma in NAFLD (George et al., 2022). Subsequently, accumulating evidence has shown that NAFLD and liver fibrosis have been linked to cardiovascular diseases, with liver fibrosis indices predicting adverse cardiovascular events and all-cause mortality in several epidemiological studies. Notably, in specific cohorts of NAFLD and Non-Alcoholic Steatohepatitis (NASH) patients, FIB-4 effectively forecasted major adverse cardiovascular events (Vieira Barbosa et al., 2022b, Anstee et al., 2024, Tang et al., 2023).

In recent years, investigations have highlighted potential connections between NAFLD, liver fibrosis, and brain health, including brain volume, brain aging, cerebral perfusion and activity, ischemic and hemorrhagic strokes, cognitive impairments, and neurodegeneration (Lombardi et al., 2019). A cross-sectional study based on the Framingham Study illustrated that NAFLD significantly correlated to smaller total cerebral brain volume, equating the difference to 4.2 years of brain aging in the general populace and 7.3 years in individuals under 60 years old (Weinstein et al., 2018). Another study based on the Rotterdam Study proved that liver steatosis and fibrosis were independently associated with decreases cerebral blood flow and brain perfusion (Yilmaz et al., 2023). Furthermore, many studies demonstrated an inconsistent association of NAFLD and liver fibrosis with stroke. A cohort study involving 9088 subjects without a history of stroke demonstrated the association between liver fibrosis and the incidence of stroke among middle-aged populations in China (Shengjun et al., 2022). Another study found that liver fibrosis was an independent predictor of long-term all-cause and cardiovascular mortality in patients with ischemic stroke (Baik et al., 2019a). Nevertheless, some other studies failed to find an association between NAFLD and liver fibrosis and stroke (Lombardi et al., 2019; Wang et al., 2022). Baik et al. even found that more severe hepatic steatosis protected ischemic stroke (Baik et al., 2019b).

Due to similar pathological mechanisms such as insulin resistance (IR), oxidative stress, and inflammation, NAFLD and liver fibrosis were considered to be associated with neurodegenerative diseases, encompassing cognitive impairment, dementia, and PD (Lombardi et al., 2019). An early study by Elliott et al. was among the first to propose and confirm the potential link between cognitive impairment and NAFLD (Colognesi et al., 2020). Subsequently, several observations have validated the association. Recently, Parikh et al. investigated the association of liver fibrosis with dementia and cognitive impairment in the UK Biobank study, uncovering that liver fibrosis in midlife significantly elevated the risk of subsequent dementia (Parikh et al., 2022). Then, they further examined cognitive tests and brain imaging data and proved that liver fibrosis was associated with worse Digit Symbol Substitution Test (DSST) and executive function-related assessments but not memory (Parikh et al., 2023). Contrarily, some other studies failed to reach the same result or even completely opposite conclusions. Xiao et al. conducted a cross-sectional and longitudinal study in the Rotterdam Study. They found neither NAFLD nor liver fibrosis increased the risk of dementia and cognitive impairment, but rather a protective effect on cognition during the first 5 years of follow-up (Xiao et al., 2022). Studies on the correlation between NAFLD, liver fibrosis, and PD were relatively scarce and yielded inconsistent results. A national cohort study in Israel found that non-alcoholic steatohepatitis (NASH) could heighten the risk of PD, whereas a subsequent large cohort study in Korea indicated gender-specific differences, with NAFLD increasing PD risk in women but decreasing it in men (Goldstein et al., 2019; Jeong et al., 2021). However, a recent cohort study by Laurens et al. based on the Rotterdam study failed to find an association between fatty liver and PD in the European population, regardless of gender (van Kleef et al., 2023). The above studies on NAFLD and PD have reached inconsistent conclusions. Very recently, a cohort study reported that liver fibrosis was associated with a decline in multiple cognitive domains in patients with PD, aligning with our findings (Zolin et al., 2024). However, we explored the correlation between liver fibrosis and PD more comprehensively, and for the first time, found a correlation between baseline FIB-4 and the longitudinal changes in the anxiety scale (STAI). Additionally, we found baseline FIB-4 levels to be associated with daytime sleepiness, motor dysfunction, cognitive impairment at baseline, and daytime sleepiness at the five-year follow-up time-point. However, due to the lack of scales related to social function, the correlation between FIB-4 and social function of PD, a field that has received great attention in recent years, is not clear (Perepezko et al., 2019; Su et al., 2020)

The liver is the main organ in the human body for energy balance and metabolism of toxic compounds. Thus, hepatic disorders could lead to inhibited clearance of toxic and harmful substances, potentially connecting liver and neurodegeneration through the known brain-liver axis (Vegas-Suárez et al., 2022). However, the underlying pathophysiological mechanisms of the correlation between NAFLD or liver fibrosis and PD remain unclear. We speculate that factors such as insulin resistance (IR), neuroinflammation, gut microbiota disorder, and neurotoxin accumulation might play significant roles (Lombardi et al., 2019; Vegas-Suárez et al., 2022). IR is a hallmark of NAFLD and liver fibrosis, which can accelerate the accumulation of liver fat and promote the release of inflammatory substances (Lade et al., 2014). IR could also promote alpha-synuclein accumulation and disrupt insulin signaling in dopaminergic neurons, leading to dopaminergic dysfunction, reduced mitochondrial oxidative activity, and ultimately, the onset and progression of PD (Athauda and Foltynie, 2016). Besides, NAFLD and liver fibrosis are often characterized by a proinflammatory state, which is also involved in the pathogenesis of PD (Schuster et al., 2018). Neuroinflammation is a common feature that accompanies liver fibrosis, which leads to the reactivity of microglia and increases the synthesis of other proinflammatory cytokines, promotes the recruitment of monocytes, and even alters the blood-brain barrier (BBB) permeability, thereby promoting the degeneration of dopamine neurons and contributing to the occurrence and development of PD (Zimmermann and Brockmann, 2022). In addition, alterations in the gut microbiota and increased intestinal permeability associated with NAFLD and fibrosis may promote neuroinflammation and alpha-synuclein accumulation, exacerbating PD progression (Tilg et al., 2022; Sun and Shen, 2018). Moreover, reduced neurotoxin clearance and altered neurotransmitter activity linked to these liver conditions might also contribute to the development of PD (Vegas-Suárez et al., 2022). To further understand the mechanisms underlying the association between NFLPD or liver fibrosis and PD, further research is needed.

Our study contributed to the evidence for the association of liver fibrosis with PD, and further investigation into the underlying mechanisms is crucial as it could unveil new targets and approaches for the early treatment of PD. However, some limitations of our study should be noted. First, because of strict inclusion and exclusion criteria, we included only patients with complete data on all clinical scales at baseline and at the 5-year follow-up time-point, which might limit the generalizability and possibly introduce bias. Second, many patients had missing assessment data at various time points, which may have affected our analyses and conclusions. Third, some other confounding factors such as other underlying medical conditions, ethnicity, predispositions and genetics including GBA, microbial 16S rRNA gene, et al. were not included in the analysis. (Qian et al., 2018; Chang et al., 2024). Forth, the dynamic changes of FIB-4 were ignored because ALT and AST may change dynamically with time. At last, this study lack the histological and imaging diagnosis of NAFLD and liver fibrosis, and it must be acknowledged that the predictive value of FIB-4 for liver fibrosis may not be ideal in some age groups (Lai et al., 2024).

## 5 Conclusion

In conclusion, this study has conducted an extensive examination of the relationship between liver fibrosis and Parkinson's Disease (PD) through the lens of the FIB-4 marker. Our data reveals that baseline FIB-4 level was associated with daytime sleepiness, motor difficulties, and cognitive impairments at baseline. Besides, patients presenting higher baseline FIB-4 levels exhibited a more rapid progression in multiple cognitive subdomains, including executive function, information processing ability, and memory. Interestingly, a higher baseline FIB-4 also seemed to convey a potential protective effect against anxiety, hinting at a multifaceted link between liver fibrosis and the progression of PD. Further research is necessary to validate these results and to delve deeper into the underlying mechanisms.

## Data Availability

Publicly available datasets were analyzed in this study. This data can be found at: https://www.ppmi-info.org/.

## Footnotes

^1^ https://www.ppmi-info.org

^2^ http://www.ppmi-info.org/study-design

^3^ http://ppmi-info.org
