# Supplementary material for: Baseline liver fibrosis-4 score correlates to the progression of anxiety and cognitive impairment in patients with Parkinson’s disease
Source: Front Aging Neurosci. 2025 Jan 24;17:1501319. doi: 10.3389/fnagi.2025.1501319 (PMC11802528; doi:10.3389/fnagi.2025.1501319)
Supplement: Supplementary file 1 [file Table_1.DOCX]

Supplementary Material

# Supplementary Tables

**Supplementary** **Table 1.** Comparison of the longitudinal change of clinical scale scores in different FIB-4 subgroups over 5 years using general linear mixed-effect models

| **Clinical scales** | Low FIB-4 subgroup | High FIB-4 subgroup | Difference in Low FIB-4 subgroup(95% CI) | Difference in high FIB-4 subgroup(95% CI) | Difference between subgroups(95% CI) | P^a^ | P^b^ |
| --- | --- | --- | --- | --- | --- | --- | --- |
| MoCA, estimated mean (SD) 0.364 | | | | | | | |
| Enrollment | 27.58 (0.45) | 27.95 (0.46) | - | - | 0.37 (-0.19, 0.94) | 0.199 |  |
| 1 | 26.97 (0.45) | 27.10 (0.46) | -0.61 (-1.00, -0.23) | -0.85 (-1.28, -0.43) | -0.24 (-0.81, 0.34) | 0.417 |  |
| 2 | 26.84 (0.45) | 27.24 (0.46) | -0.74 (-1.13, -0.36) | -0.71 (-1.14, -0.29) | 0.03 (-0.54, 0.60) | 0.914 |  |
| 3 | 27.09 (0.45) | 27.29 (0.46) | -0.49 (-0.87, -0.11) | -0.66 (-1.08, -0.25) | -0.17 (-0.74, 0.39) | 0.548 |  |
| 4 | 27.15 (0.45) | 27.22 (0.46) | -0.43 (-0.81, -0.06) | -0.73 (-1.15, -0.32) | -0.30 (-0.86, 0.26) | 0.296 |  |
| 5 | 27.31 (0.45) | 27.14 (0.46) | -0.27 (-0.64, 0.11) | -0.81 (-1.23, -0.40) | -0.55 (-1.10, 0.01) | 0.055 |  |
| UPDRSI, estimated mean (SD) 0.041 | | | | | | | |
| Enrollment | 0.96 (0.32) | 0.81 (0.33) | - | - | -0.15 (-0.59, 0.28) | 0.492 |  |
| 1 | 1.03 (0.32) | 1.18 (0.33) | 0.07 (-0.25, 0.40) | 0.38 (0.02, 0.74) | 0.30 (-0.18, 0.79) | 0.216 |  |
| 2 | 1.10 (0.32) | 1.56 (0.33) | 0.14 (-0.18, 0.46) | 0.75 (0.39, 1.10) | 0.61 (0.13, 1.09) | 0.013 |  |
| 3 | 1.30 (0.32) | 1.48 (0.33) | 0.34 (0.02, 0.66) | 0.68 (0.33, 1.03) | 0.34 (-0.14, 0.81) | 0.162 |  |
| 4 | 1.35 (0.32) | 1.95 (0.33) | 0.39 (0.07, 0.71) | 1.14 (0.79, 1.49) | 0.75 (0.28, 1.22) | 0.002 |  |
| 5 | 1.72 (0.32) | 2.00 (0.33) | 0.76 (0.44, 1.08) | 1.20 (0.85, 1.54) | 0.44 (-0.04, 0.91) | 0.070 |  |
| UPDRSII, estimated mean (SD) 0.801 | | | | | | | |
| Enrollment | 4.78 (0.81) | 4.66 (0.83) | - | - | -0.12 (-1.15, 0.91) | 0.817 |  |
| 1 | 5.96 (0.81) | 6.00 (0.84) | 1.18 (0.47, 1.88) | 1.34 (0.56, 2.12) | 0.17 (-0.88, 1.22) | 0.756 |  |
| 2 | 6.48 (0.81) | 7.08 (0.84) | 1.70 (1.00, 2.40) | 2.43 (1.65, 3.20) | 0.73 (-0.32, 1.77) | 0.173 |  |
| 3 | 7.60 (0.81) | 7.73 (0.83) | 2.82 (2.13, 3.51) | 3.08 (2.31, 3.84) | 0.26 (-0.78, 1.29) | 0.626 |  |
| 4 | 8.30 (0.81) | 8.70 (0.83) | 3.52 (2.83, 4.21) | 4.04 (3.28, 4.81) | 0.52 (-0.51, 1.55) | 0.322 |  |
| 5 | 8.92 (0.81) | 9.15 (0.83) | 4.14 (3.45, 4.83) | 4.49 (3.73, 5.25) | 0.35 (-0.68, 1.37) | 0.506 |  |
| UPDRSIII, estimated mean (SD) 0.493 | | | | | | | |
| Enrollment | 15.88 (1.93) | 15.87 (1.99) | - | - | -0.01 (-2.45, 2.43) | 0.992 |  |
| 1 | 18.87 (1.94) | 19.09 (2.01) | 2.99 (1.36, 4.62) | 3.22 (1.40, 5.04) | 0.23 (-2.21, 2.68) | 0.853 |  |
| 2 | 20.19 (1.93) | 21.24 (2.00) | 4.30 (2.67, 5.93) | 5.37 (3.57, 7.17) | 1.07 (-1.36, 3.50) | 0.389 |  |
| 3 | 23.18 (1.93) | 22.47 (2.00) | 7.30 (5.69, 8.91) | 6.60 (4.82, 8.39); p<0.001 | -0.70 (-3.10, 1.70) | 0.569 |  |
| 4 | 25.71 (1.93) | 24.75 (2.00) | 9.83 (8.22, 11.43) | 8.88 (7.10, 10.66) | -0.95 (-3.34, 1.45) | 0.438 |  |
| 5 | 26.56 (1.93) | 25.46 (1.99) | 10.68 (9.08, 12.28) | 9.59 (7.82, 11.35) | -1.09 (-3.47, 1.28) | 0.367 |  |
| STAI-State subscore, estimated mean (SD) 0.018 | | | | | | | |
| Enrollment | 47.75 (0.78) | 48.37 (0.82) | - | - | 0.62 (-0.54, 1.79) | 0.294 |  |
| 1 | 47.73 (0.79) | 47.12 (0.82) | -0.02 (-0.98, 0.94) | -1.25 (-2.31, -0.20) | -1.23 (-2.66, 0.19) | 0.089 |  |
| 2 | 48.07 (0.79) | 46.37 (0.82) | 0.32 (-0.63, 1.28) | -2.00 (-3.05, -0.96) | -2.33 (-3.74, -0.91) | 0.001 |  |
| 3 | 48.34 (0.79) | 47.21 (0.82) | 0.59 (-0.35, 1.53) | -1.17 (-2.21, -0.13) | -1.76 (-3.16, -0.36) | 0.014 |  |
| 4 | 48.02 (0.79) | 46.77 (0.82) | 0.27 (-0.67, 1.21) | -1.60 (-2.64, -0.56) | -1.87 (-3.27, -0.47) | 0.009 |  |
| 5 | 48.10 (0.78) | 46.70 (0.82) | 0.35 (-0.59, 1.28) | -1.67 (-2.70, -0.64) | -2.02 (-3.41, -0.63) | 0.004 |  |
| STAI-Trait subscore, estimated mean (SD) <0.001 | | | | | | | |
| Enrollment | 45.48 (0.66) | 45.96 (0.68) | - | - | 0.48 (-0.46, 1.42) | 0.314 |  |
| 1 | 44.78 (0.66) | 45.73 (0.69) | -0.70 (-1.44, 0.04) | -0.24 (-1.05, 0.57) | 0.46 (-0.63, 1.56) | 0.407 |  |
| 2 | 45.65 (0.66) | 44.74 (0.69) | 0.17 (-0.56, 0.91) | -1.22 (-2.03, -0.41) | -1.39 (-2.48, -0.30) | 0.013 |  |
| 3 | 45.80 (0.66) | 44.83 (0.69) | 0.32 (-0.40, 1.05) | -1.13 (-1.93, -0.33) | -1.45 (-2.53, -0.37) | 0.008 |  |
| 4 | 45.18 (0.66) | 44.82 (0.69) | -0.31 (-1.03, 0.42) | -1.14 (-1.95, -0.34) | -0.84 (-1.92, 0.24) | 0.129 |  |
| 5 | 45.52 (0.66) | 44.46 (0.68) | 0.04 (-0.68, 0.76) | -1.50 (-2.30, -0.71) | -1.54 (-2.62, -0.47) | 0.005 |  |
| STAI-Total, estimated mean (SD) <0.001 | | | | | | | |
| Enrollment | 93.16 (1.22) | 94.11 (1.27) | - | - | 0.96 (-0.79, 2.70) | 0.283 |  |
| 1 | 92.43 (1.23) | 92.61 (1.28) | -0.73 (-2.11, 0.65) | -1.50 (-3.02, 0.02) | -0.77 (-2.82, 1.28) | 0.461 |  |
| 2 | 93.65 (1.23) | 90.88 (1.27) | 0.49 (-0.88, 1.87) | -3.23 (-4.74, -1.72) | -3.72 (-5.76, -1.68) | <0.001 |  |
| 3 | 94.07 (1.22) | 91.82 (1.27) | 0.92 (-0.44, 2.27) | -2.29 (-3.79, -0.80) | -3.21 (-5.23, -1.19) | 0.002 |  |
| 4 | 93.12 (1.22) | 91.36 (1.27) | -0.04 (-1.39, 1.32) | -2.75 (-4.25, -1.25) | -2.71 (-4.73, -0.69) | 0.009 |  |
| 5 | 93.54 (1.22) | 90.93 (1.27) | 0.39 (-0.96, 1.73) | -3.18 (-4.66, -1.69) | -3.57 (-5.57, -1.56) | <0.001 |  |
| QUIP, estimated mean (SD) 0.241 | | | | | | | |
| Enrollment | 3.60 (0.27) | 3.42 (0.28) | - | - | -0.18 (-0.59, 0.23) | 0.384 |  |
| 1 | 1.77 (0.27) | 1.37 (0.28) | -1.83 (-2.17, -1.49) | -2.04 (-2.42, -1.67) | -0.21 (-0.72, 0.30) | 0.412 |  |
| 2 | 0.91 (0.27) | 0.85 (0.28) | -2.68 (-3.03, -2.34) | -2.56 (-2.94, -2.19) | 0.12 (-0.39, 0.63) | 0.642 |  |
| 3 | 0.66 (0.27) | 0.67 (0.28) | -2.94 (-3.28, -2.60) | -2.75 (-3.12, -2.38) | 0.19 (-0.31, 0.69) | 0.457 |  |
| 4 | 0.60 (0.27) | 0.79 (0.28) | -2.99 (-3.33, -2.66) | -2.63 (-3.00, -2.25) | 0.37 (-0.13, 0.87) | 0.151 |  |
| 5 | 0.92 (0.27) | 0.61 (0.28) | -2.68 (-3.01, -2.35) | -2.80 (-3.17, -2.44) | -0.12 (-0.62, 0.37) | 0.623 |  |
| GDS, estimated mean (SD) 0.392 | | | | | | | |
| Enrollment | 5.28 (0.21) | 5.17 (0.22) | - | - | -0.11 (-0.47, 0.25) | 0.549 |  |
| 1 | 5.13 (0.21) | 5.49 (0.22) | -0.15 (-0.46, 0.17) | 0.33 (-0.03, 0.68) | 0.47 (-0.00, 0.94) | 0.051 |  |
| 2 | 5.31 (0.21) | 5.50 (0.22) | 0.04 (-0.29, 0.36) | 0.33 (-0.03, 0.69) | 0.29 (-0.19, 0.77) | 0.234 |  |
| 3 | 5.30 (0.21) | 5.53 (0.22) | 0.03 (-0.29, 0.34) | 0.36 (0.01, 0.71) | 0.33 (-0.14, 0.80) | 0.170 |  |
| 4 | 5.36 (0.21) | 5.40 (0.22) | 0.08 (-0.24, 0.40) | 0.24 (-0.11, 0.59) | 0.16 (-0.32, 0.63) | 0.519 |  |
| 5 | 5.48 (0.21) | 5.45 (0.22) | 0.20 (-0.12, 0.52) | 0.28 (-0.07, 0.64) | 0.08 (-0.39, 0.56) | 0.726 |  |
| SCOPA, estimated mean (SD) 0.305 | | | | | | | |
| Enrollment | 12.86 (1.48) | 12.25 (1.53) | - | - | -0.61 (-2.53, 1.31) | 0.531 |  |
| 1 | 14.56 (1.49) | 13.82 (1.54) | 1.70 (0.37, 3.04) | 1.58 (0.09, 3.06) | -0.13 (-2.12, 1.87) | 0.901 |  |
| 2 | 15.20 (1.49) | 15.16 (1.54) | 2.34 (1.00, 3.67) | 2.92 (1.45, 4.38) | 0.58 (-1.41, 2.56) | 0.567 |  |
| 3 | 16.13 (1.49) | 16.08 (1.53) | 3.27 (1.96, 4.59) | 3.83 (2.38, 5.28) | 0.56 (-1.40, 2.52) | 0.577 |  |
| 4 | 16.04 (1.49) | 17.45 (1.53) | 3.18 (1.86, 4.49) | 5.21 (3.75, 6.66) | 2.03 (0.07, 3.99) | 0.043 |  |
| 5 | 17.79 (1.48) | 18.21 (1.53) | 4.93 (3.62, 6.23) | 5.96 (4.52, 7.40) | 1.03 (-0.91, 2.98) | 0.298 |  |
| EPSS, estimated mean (SD) 0.764 | | | | | | | |
| Enrollment | 5.26 (0.73) | 5.61 (0.76) | - | - | 0.34 (-0.59, 1.27) | 0.469 |  |
| 1 | 5.65 (0.73) | 6.20 (0.76) | 0.38 (-0.24, 1.00) | 0.59 (-0.10, 1.28) | 0.21 (-0.71, 1.13) | 0.657 |  |
| 2 | 6.55 (0.73) | 6.58 (0.76) | 1.29 (0.67, 1.91) | 0.97 (0.29, 1.65) | -0.32 (-1.24, 0.60) | 0.497 |  |
| 3 | 7.09 (0.73) | 7.20 (0.76) | 1.83 (1.22, 2.44) | 1.60 (0.93, 2.27) | -0.23 (-1.14, 0.68) | 0.623 |  |
| 4 | 7.26 (0.73) | 7.19 (0.76) | 1.99 (1.39, 2.60) | 1.58 (0.91, 2.26) | -0.41 (-1.32, 0.50) | 0.373 |  |
| 5 | 7.71 (0.73) | 7.69 (0.76) | 2.45 (1.84, 3.05) | 2.09 (1.42, 2.76) | -0.36 (-1.26, 0.54) | 0.437 |  |
| RBDSQ, estimated mean (SD) 0.734 | | | | | | | |
| Enrollment | 3.54 (0.45) | 3.37 (0.47) | - | - | -0.17 (-0.75, 0.40) | 0.554 |  |
| 1 | 3.34 (0.45) | 3.26 (0.47) | -0.20 (-0.59, 0.19) | -0.11 (-0.54, 0.33) | 0.10 (-0.49, 0.68) | 0.746 |  |
| 2 | 3.97 (0.45) | 3.84 (0.47) | 0.42 (0.03, 0.82) | 0.47 (0.04, 0.91) | 0.05 (-0.53, 0.63) | 0.866 |  |
| 3 | 4.04 (0.45) | 4.29 (0.47) | 0.49 (0.11, 0.88) | 0.92 (0.50, 1.35) | 0.43 (-0.14, 1.01) | 0.142 |  |
| 4 | 4.31 (0.45) | 4.22 (0.47) | 0.76 (0.38, 1.15) | 0.85 (0.42, 1.28) | 0.09 (-0.49, 0.67) | 0.760 |  |
| 5 | 4.43 (0.45) | 4.47 (0.47) | 0.89 (0.50, 1.27) | 1.10 (0.67, 1.52) | 0.21 (-0.36, 0.78) | 0.470 |  |
| ADL, estimated mean (SD) 0.332 | | | | | | | |
| Enrollment | 95.09 (1.40) | 95.18 (1.45) | - | - | 0.08 (-1.76, 1.93) | 0.928 |  |
| 1 | 92.20 (1.41) | 92.81 (1.46) | -2.90 (-4.22, -1.57) | -2.37 (-3.84, -0.89) | 0.53 (-1.46, 2.51) | 0.604 |  |
| 2 | 91.54 (1.41) | 90.16 (1.45) | -3.55 (-4.88, -2.22) | -5.02 (-6.48, -3.56) | -1.47 (-3.44, 0.51) | 0.146 |  |
| 3 | 90.03 (1.40) | 89.66 (1.45) | -5.07 (-6.38, -3.76) | -5.52 (-6.97, -4.08) | -0.46 (-2.41, 1.49) | 0.647 |  |
| 4 | 88.96 (1.40) | 87.67 (1.45) | -6.14 (-7.45, -4.83) | -7.51 (-8.96, -6.07) | -1.37 (-3.32, 0.58) | 0.167 |  |
| 5 | 87.09 (1.40) | 86.60 (1.45) | -8.00 (-9.30, -6.70) | -8.58 (-10.01, -7.14) | -0.58 (-2.51, 1.36) | 0.559 |  |
| BJOL, estimated mean (SD) 0.108 | | | | | | | |
| Enrollment | 13.12 (0.32) | 13.24 (0.33) | - | - | 0.13 (-0.31, 0.56) | 0.569 |  |
| 1 | 12.67 (0.32) | 12.92 (0.33) | -0.44 (-0.76, -0.12) | -0.32 (-0.68, 0.04) | 0.12 (-0.36, 0.61) | 0.611 |  |
| 2 | 13.13 (0.32) | 13.23 (0.33) | 0.01 (-0.31, 0.33) | -0.01 (-0.36, 0.34) | -0.02 (-0.50, 0.46) | 0.942 |  |
| 3 | 12.85 (0.32) | 13.07 (0.33) | -0.27 (-0.59, 0.05) | -0.18 (-0.53, 0.17) | 0.09 (-0.38, 0.57) | 0.700 |  |
| 4 | 13.26 (0.32) | 13.03 (0.33) | 0.14 (-0.17, 0.46) | -0.21 (-0.57, 0.14) | -0.36 (-0.83, 0.12) | 0.141 |  |
| 5 | 12.80 (0.32) | 12.51 (0.33) | -0.32 (-0.63, -0.01) | -0.73 (-1.08, -0.38) | -0.41 (-0.88, 0.06) | 0.085 |  |
| LSNT, estimated mean (SD) 0.005 | | | | | | | |
| Enrollment | 10.96 (0.37) | 11.10 (0.39) | - | - | 0.14 (-0.36, 0.64) | 0.579 |  |
| 1 | 10.75 (0.38) | 11.21 (0.39) | -0.21 (-0.57, 0.15) | 0.11 (-0.30, 0.51) | 0.32 (-0.23, 0.86) | 0.252 |  |
| 2 | 10.88 (0.38) | 10.96 (0.39) | -0.08 (-0.44, 0.29) | -0.14 (-0.54, 0.26) | -0.06 (-0.60, 0.48) | 0.821 |  |
| 3 | 10.75 (0.38) | 10.55 (0.39) | -0.21 (-0.57, 0.15) | -0.55 (-0.94, -0.15) | -0.34 (-0.87, 0.19) | 0.211 |  |
| 4 | 10.88 (0.37) | 10.50 (0.39) | -0.08 (-0.44, 0.28) | -0.60 (-1.00, -0.21) | -0.52 (-1.06, 0.01) | 0.054 |  |
| 5 | 10.61 (0.37) | 10.12 (0.39) | -0.35 (-0.71, 0.00) | -0.98 (-1.38, -0.59) | -0.63 (-1.16, -0.10) | 0.019 |  |
| SDMT, estimated mean (SD) 0.007 | | | | | | | |
| Enrollment | 43.89 (1.46) | 44.25 (1.51) | - | - | 0.36 (-1.53, 2.25) | 0.711 |  |
| 1 | 43.99 (1.47) | 43.78 (1.52) | 0.10 (-1.22, 1.41) | -0.47 (-1.93, 0.99) | -0.57 (-2.53, 1.40) | 0.574 |  |
| 2 | 43.38 (1.47) | 42.45 (1.51) | -0.51 (-1.83, 0.81) | -1.80 (-3.25, -0.35) | -1.29 (-3.24, 0.67) | 0.198 |  |
| 3 | 43.05 (1.46) | 42.49 (1.51) | -0.84 (-2.14, 0.46) | -1.76 (-3.19, -0.32) | -0.92 (-2.86, 1.02) | 0.353 |  |
| 4 | 42.93 (1.46) | 40.80 (1.51) | -0.96 (-2.26, 0.34) | -3.45 (-4.89, -2.01) | -2.49 (-4.43, -0.55) | 0.012 |  |
| 5 | 43.46 (1.46) | 40.51 (1.51) | -0.43 (-1.72, 0.86) | -3.74 (-5.16, -2.32) | -3.31 (-5.23, -1.39) | 0.001 |  |
| SFT, estimated mean (SD) 0.026 | | | | | | | |
| Enrollment | 50.32 (1.64) | 51.22 (1.70) | - | - | 0.90 (-1.29, 3.08) | 0.421 |  |
| 1 | 51.21 (1.65) | 51.19 (1.71) | 0.88 (-0.71, 2.48) | -0.03 (-1.81, 1.74) | -0.92 (-3.30, 1.47) | 0.452 |  |
| 2 | 51.31 (1.65) | 50.41 (1.70) | 0.98 (-0.61, 2.58) | -0.81 (-2.57, 0.94) | -1.80 (-4.17, 0.58) | 0.138 |  |
| 3 | 52.69 (1.64) | 50.73 (1.70) | 2.37 (0.79, 3.95) | -0.50 (-2.23, 1.24) | -2.87 (-5.21, -0.52) | 0.017 |  |
| 4 | 52.15 (1.64) | 50.22 (1.70) | 1.83 (0.25, 3.40) | -1.00 (-2.75, 0.74) | -2.83 (-5.18, -0.48) | 0.018 |  |
| 5 | 52.97 (1.64) | 50.29 (1.70) | 2.65 (1.08, 4.21) | -0.93 (-2.65, 0.80) | -3.57 (-5.90, -1.25) | 0.003 |  |
| HVLTTotalRecal, estimated mean (SD) <0.001 | | | | | | | |
| Enrollment | 45.79 (1.72) | 46.23 (1.78) | - | - | 0.44 (-1.87, 2.74) | 0.710 |  |
| 1 | 44.64 (1.73) | 44.82 (1.79) | -1.15 (-2.83, 0.53) | -1.41 (-3.28, 0.46) | -0.26 (-2.77, 2.25) | 0.840 |  |
| 2 | 46.12 (1.73) | 44.34 (1.79) | 0.33 (-1.35, 2.02) | -1.89 (-3.74, -0.04) | -2.22 (-4.72, 0.28) | 0.081 |  |
| 3 | 49.66 (1.73) | 45.97 (1.78) | 3.87 (2.20, 5.53) | -0.26 (-2.09, 1.57) | -4.12 (-6.59, -1.65) | 0.001 |  |
| 4 | 47.70 (1.73) | 43.77 (1.79) | 1.91 (0.25, 3.57) | -2.46 (-4.30, -0.62) | -4.36 (-6.84, -1.89) | 0.001 |  |
| 5 | 49.22 (1.72) | 45.79 (1.78) | 3.43 (1.79, 5.08) | -0.44 (-2.26, 1.38) | -3.87 (-6.32, -1.42) | 0.002 |  |
| HVLTDelayedRecall, estimated mean (SD) 0.033 | | | | | | | |
| Enrollment | 44.42 (1.75) | 45.33 (1.81) | - | - | 0.91 (-1.48, 3.30) | 0.455 |  |
| 1 | 44.55 (1.75) | 44.40 (1.82) | 0.13 (-1.67, 1.94) | -0.93 (-2.93, 1.07) | -1.06 (-3.75, 1.64) | 0.441 |  |
| 2 | 46.29 (1.75) | 44.87 (1.82) | 1.87 (0.07, 3.67) | -0.46 (-2.44, 1.52) | -2.33 (-5.01, 0.35) | 0.088 |  |
| 3 | 47.21 (1.75) | 45.02 (1.81) | 2.79 (1.01, 4.57) | -0.31 (-2.27, 1.65) | -3.10 (-5.75, -0.45) | 0.022 |  |
| 4 | 46.72 (1.75) | 43.52 (1.81) | 2.30 (0.52, 4.08) | -1.81 (-3.78, 0.17) | -4.11 (-6.76, -1.45) | 0.002 |  |
| 5 | 48.50 (1.75) | 46.42 (1.81) | 4.09 (2.32, 5.85) | 1.10 (-0.85, 3.05) | -2.99 (-5.62, -0.36) | 0.026 |  |
| HVLTRetention, estimated mean (SD) 0.442 | | | | | | | |
| Enrollment | 47.36 (1.70) | 48.46 (1.77) | - | - | 1.10 (-1.55, 3.76) | 0.415 |  |
| 1 | 47.26 (1.71) | 48.00 (1.79) | -0.10 (-2.38, 2.18) | -0.47 (-3.00, 2.06) | -0.37 (-3.77, 3.03) | 0.832 |  |
| 2 | 49.56 (1.71) | 49.12 (1.78) | 2.20 (-0.08, 4.48) | 0.66 (-1.85, 3.16) | -1.54 (-4.93, 1.84) | 0.371 |  |
| 3 | 47.74 (1.71) | 48.59 (1.78) | 0.38 (-1.87, 2.63) | 0.12 (-2.36, 2.60) | -0.25 (-3.60, 3.09) | 0.882 |  |
| 4 | 47.96 (1.70) | 46.10 (1.78) | 0.60 (-1.64, 2.84) | -2.37 (-4.86, 0.13) | -2.97 (-6.32, 0.39) | 0.083 |  |
| 5 | 50.19 (1.70) | 49.18 (1.77) | 2.83 (0.60, 5.06) | 0.72 (-1.75, 3.18) | -2.11 (-5.43, 1.21) | 0.213 |  |
| HVLTRecognitionDiscrimination, estimated mean (SD) 0.078 | | | | | | | |
| Enrollment | 45.68 (1.56) | 46.30 (1.63) | - | - | 0.63 (-1.74, 2.99) | 0.602 |  |
| 1 | 46.23 (1.59) | 45.97 (1.64) | 0.55 (-1.44, 2.55) | -0.33 (-2.54, 1.88) | -0.88 (-3.86, 2.09) | 0.560 |  |
| 2 | 49.61 (1.58) | 48.31 (1.64) | 3.93 (1.94, 5.92) | 2.01 (-0.18, 4.19) | -1.93 (-4.88, 1.03) | 0.201 |  |
| 3 | 48.07 (1.57) | 46.22 (1.63) | 2.40 (0.44, 4.36) | -0.08 (-2.25, 2.09) | -2.48 (-5.41, 0.45) | 0.097 |  |
| 4 | 47.73 (1.57) | 44.84 (1.64) | 2.05 (0.09, 4.01) | -1.46 (-3.65, 0.72) | -3.52 (-6.45, -0.58) | 0.019 |  |
| 5 | 49.00 (1.56) | 45.78 (1.63) | 3.32 (1.38, 5.26) | -0.52 (-2.68, 1.63) | -3.84 (-6.75, -0.94) | 0.009 |  |

Abbreviations: FIB-4: Fibrosis-4; UPDRS I, II, III: Part I, II, III of Unified Parkinson's Disease Rating Scale; GDS: Geriatric Depression Scale; STAI: State–Trait Anxiety Inventory; QUIP: Questionnaire for Impulsive-Compulsive Disorders in Parkinson's Disease (QUIP); SCOPA-AUT: Scales for Outcomes in Parkinson's disease-Autonomic; EPSS: Epworth Sleepiness Scale; RBDSQ: REM sleep behavior disorder with the REM Sleep Behavior Disorder Screening Questionnaire; ADL: Schwab & England Activities of Daily Living Scale; MoCA: Montreal Cognitive Assessment; BJOLT: Benton Judgment of Line Orientation Test; LNST: Letter Number Sequencing Test; SDMT: Symbol Digit Modalities Test; SFT: Semantic Fluency Test; HVLT: Hopkins Verbal Learning Test.

P^a^：P for Difference between subgroups; P^b^:P for FIB-4 subgroup*time

**Supplementary Table 2.** Longitudinal changes of cognitive-related scale scores in different FIB-4 subgroups in female patients during 5 years

| **Clinical scales** | Low FIB-4 subgroup | High FIB-4 subgroup | Difference in Low FIB-4 subgroup(95% CI)) | Difference in high FIB-4 subgroup(95% CI) | Difference between subgroups(95% CI) | P^a^ | P^b^ |
| --- | --- | --- | --- | --- | --- | --- | --- |
| LSNT, estimated mean (SD) 0.091 | | | | | | | |
| Enrollment | 10.50 (0.55) | 10.88 (0.62) | - | - | 0.38 (-0.48, 1.24) | 0.385 |  |
| 1 | 10.54 (0.55) | 10.51 (0.62) | 0.04 (-0.53, 0.60) | -0.37 (-1.12, 0.38) | -0.41 (-1.35, 0.54) | 0.399 |  |
| 2 | 10.64 (0.55) | 10.65 (0.62) | 0.14 (-0.43, 0.70) | -0.23 (-0.98, 0.52) | -0.37 (-1.31, 0.57) | 0.444 |  |
| 3 | 10.48 (0.55) | 10.04 (0.62) | -0.01 (-0.57, 0.54) | -0.84 (-1.59, -0.10) | -0.83 (-1.76, 0.10) | 0.080 |  |
| 4 | 11.02 (0.55) | 10.15 (0.62) | 0.52 (-0.03, 1.07) | -0.73 (-1.46, 0.01) | -1.25 (-2.17, -0.32) | 0.008 |  |
| 5 | 10.64 (0.55) | 10.03 (0.62) | 0.14 (-0.42, 0.69) | -0.85 (-1.59, -0.11) | -0.98 (-1.91, -0.06) | 0.036 |  |
| SDMT, estimated mean (SD) 0.075 | | | | | | | |
| Enrollment | 46.12 (2.32) | 47.65 (2.61) | - | - | 1.52 (-1.94, 4.99) | 0.388 |  |
| 1 | 46.64 (2.33) | 46.03 (2.63) | 0.51 (-1.59, 2.62) | -1.61 (-4.39, 1.16) | -2.13 (-5.61, 1.36) | 0.231 |  |
| 2 | 46.73 (2.33) | 45.37 (2.63) | 0.61 (-1.47, 2.69) | -2.28 (-5.06, 0.50) | -2.89 (-6.36, 0.58) | 0.102 |  |
| 3 | 46.32 (2.32) | 45.31 (2.63) | 0.19 (-1.86, 2.24) | -2.34 (-5.11, 0.44) | -2.53 (-5.98, 0.92) | 0.151 |  |
| 4 | 45.34 (2.32) | 42.38 (2.61) | -0.78 (-2.83, 1.27) | -5.27 (-8.00, -2.55) | -4.49 (-7.90, -1.08) | 0.010 |  |
| 5 | 46.87 (2.32) | 43.62 (2.61) | 0.75 (-1.29, 2.78) | -4.03 (-6.76, -1.30) | -4.78 (-8.18, -1.37) | 0.006 |  |
| SFT, estimated mean (SD) 0.071 | | | | | | | |
| Enrollment | 52.80 (2.57) | 54.60 (2.91) | - | - | 1.79 (-2.14, 5.73) | 0.372 |  |
| 1 | 55.27 (2.58) | 54.95 (2.92) | 2.47 (-0.02, 4.95) | 0.35 (-2.93, 3.62) | -2.12 (-6.23, 1.99) | 0.312 |  |
| 2 | 54.35 (2.58) | 53.66 (2.92) | 1.54 (-0.91, 4.00) | -0.94 (-4.22, 2.33) | -2.48 (-6.58, 1.61) | 0.234 |  |
| 3 | 56.26 (2.57) | 53.51 (2.91) | 3.45 (1.04, 5.87) | -1.09 (-4.33, 2.16) | -4.54 (-8.59, -0.50) | 0.028 |  |
| 4 | 56.81 (2.57) | 54.02 (2.91) | 4.00 (1.59, 6.42) | -0.58 (-3.79, 2.64) | -4.58 (-8.60, -0.56) | 0.026 |  |
| 5 | 58.53 (2.57) | 54.69 (2.91) | 5.73 (3.32, 8.13) | 0.09 (-3.13, 3.31) | -5.64 (-9.65, -1.62) | 0.006 |  |
| HVLT Total Recal, estimated mean (SD) 0.181 | | | | | | | |
| Enrollment | 47.64 (2.34) | 46.86 (2.68) | - | - | -0.79 (-4.65, 3.08) | 0.690 |  |
| 1 | 45.86 (2.36) | 45.57 (2.70) | -1.79 (-4.49, 0.92) | -1.29 (-4.85, 2.28) | 0.50 (-3.97, 4.97) | 0.827 |  |
| 2 | 47.83 (2.35) | 43.88 (2.70) | 0.18 (-2.49, 2.85) | -2.98 (-6.54, 0.59) | -3.16 (-7.62, 1.29) | 0.164 |  |
| 3 | 51.19 (2.35) | 47.63 (2.68) | 3.54 (0.91, 6.18) | 0.77 (-2.77, 4.30) | -2.78 (-7.18, 1.63) | 0.216 |  |
| 4 | 50.11 (2.35) | 45.65 (2.68) | 2.47 (-0.16, 5.10) | -1.21 (-4.71, 2.29) | -3.68 (-8.06, 0.70) | 0.099 |  |
| 5 | 50.51 (2.34) | 45.53 (2.68) | 2.86 (0.25, 5.48) | -1.33 (-4.83, 2.17) | -4.20 (-8.57, 0.17) | 0.060 |  |
| HVLT Delayed Recall, estimated mean (SD) 0.989 | | | | | | | |
| Enrollment | 45.28 (2.49) | 45.66 (2.83) | - | - | 0.38 (-3.65, 4.41) | 0.853 |  |
| 1 | 45.03 (2.51) | 46.00 (2.85) | -0.25 (-3.03, 2.53) | 0.34 (-3.33, 4.01) | 0.59 (-4.01, 5.20) | 0.801 |  |
| 2 | 46.18 (2.50) | 45.48 (2.85) | 0.90 (-1.85, 3.66) | -0.17 (-3.84, 3.50) | -1.08 (-5.67, 3.51) | 0.645 |  |
| 3 | 46.77 (2.49) | 47.51 (2.84) | 1.50 (-1.21, 4.20) | 1.85 (-1.78, 5.49) | 0.36 (-4.18, 4.89) | 0.877 |  |
| 4 | 47.34 (2.49) | 47.75 (2.83) | 2.07 (-0.64, 4.78) | 2.09 (-1.51, 5.70) | 0.02 (-4.48, 4.53) | 0.992 |  |
| 5 | 47.38 (2.49) | 47.81 (2.83) | 2.10 (-0.59, 4.80) | 2.15 (-1.45, 5.76) | 0.05 (-4.45, 4.55) | 0.983 |  |

Abbreviations: FIB-4: Fibrosis-4; LNST: Letter Number Sequencing Test; SDMT: Symbol Digit Modalities Test; SFT: Semantic Fluency Test; HVLT: Hopkins Verbal Learning Test.

P^a^：P for Difference between subgroups; P^b^:P for FIB-4 subgroup*time

**Supplementary** **Table 3.** Longitudinal changes of cognitive-related scale scores in different FIB-4 subgroups in male patients during 5 years

| **Clinical scales** | Low FIB-4 subgroup | High FIB-4 subgroup | Difference in Low FIB-4 subgroup(95% CI)) | Difference in high FIB-4 subgroup(95% CI) | Difference between subgroups(95% CI) | P^a^ | P^b^ |
| --- | --- | --- | --- | --- | --- | --- | --- |
| LSNT, estimated mean (SD) 0.079 | | | | | | | |
| Enrollment | 11.34 (0.53) | 11.31 (0.53) | - | - | -0.02 (-0.64, 0.60) | 0.941 |  |
| 1 | 10.97 (0.53) | 11.60 (0.53) | -0.37 (-0.84, 0.10) | 0.29 (-0.19, 0.76) | 0.66 (-0.01, 1.33) | 0.055 |  |
| 2 | 11.12 (0.53) | 11.21 (0.53) | -0.21 (-0.68, 0.26) | -0.10 (-0.57, 0.37) | 0.11 (-0.56, 0.78) | 0.745 |  |
| 3 | 11.01 (0.53) | 10.87 (0.53) | -0.33 (-0.80, 0.13) | -0.44 (-0.91, 0.03) | -0.11 (-0.77, 0.55) | 0.746 |  |
| 4 | 10.87 (0.53) | 10.76 (0.53) | -0.47 (-0.93, -0.00) | -0.55 (-1.02, -0.08) | -0.09 (-0.75, 0.58) | 0.800 |  |
| 5 | 10.67 (0.53) | 10.28 (0.53) | -0.67 (-1.13, -0.21) | -1.03 (-1.50, -0.57) | -0.36 (-1.02, 0.29) | 0.276 |  |
| SDMT, estimated mean (SD) 0.167 | | | | | | | |
| Enrollment | 41.85 (1.94) | 42.06 (1.92) | - | - | 0.21 (-2.05, 2.47) | 0.856 |  |
| 1 | 41.64 (1.95) | 42.02 (1.93) | -0.20 (-1.90, 1.49) | -0.04 (-1.77, 1.69) | 0.17 (-2.25, 2.58) | 0.893 |  |
| 2 | 40.58 (1.95) | 40.45 (1.92) | -1.26 (-2.97, 0.44) | -1.60 (-3.31, 0.10) | -0.34 (-2.75, 2.07) | 0.782 |  |
| 3 | 40.32 (1.95) | 40.53 (1.92) | -1.53 (-3.22, 0.16) | -1.53 (-3.21, 0.16) | 0.00 (-2.38, 2.38) | 1.000 |  |
| 4 | 40.78 (1.94) | 39.32 (1.92) | -1.07 (-2.75, 0.61) | -2.74 (-4.44, -1.03) | -1.67 (-4.06, 0.73) | 0.172 |  |
| 5 | 40.65 (1.94) | 38.42 (1.92) | -1.20 (-2.86, 0.47) | -3.63 (-5.31, -1.96) | -2.44 (-4.80, -0.08) | 0.043 |  |
| SF, estimated mean (SD) 0.607 | | | | | | | |
| Enrollment | 48.52 (2.24) | 49.03 (2.23) | - | - | 0.52 (-2.14, 3.17) | 0.704 |  |
| 1 | 48.38 (2.25) | 48.86 (2.24) | -0.14 (-2.21, 1.93) | -0.17 (-2.28, 1.94) | -0.04 (-2.99, 2.92) | 0.981 |  |
| 2 | 49.15 (2.25) | 48.27 (2.23) | 0.63 (-1.45, 2.72) | -0.76 (-2.84, 1.33) | -1.39 (-4.33, 1.56) | 0.356 |  |
| 3 | 50.18 (2.24) | 48.75 (2.23) | 1.66 (-0.40, 3.73) | -0.28 (-2.34, 1.78) | -1.94 (-4.86, 0.97) | 0.192 |  |
| 4 | 48.94 (2.24) | 47.88 (2.23) | 0.42 (-1.64, 2.48) | -1.15 (-3.23, 0.93) | -1.57 (-4.50, 1.35) | 0.292 |  |
| 5 | 49.16 (2.24) | 47.73 (2.23) | 0.65 (-1.39, 2.68) | -1.30 (-3.35, 0.75) | -1.95 (-4.84, 0.94) | 0.186 |  |
| HVLT Total Recal, estimated mean (SD) 0.005 | | | | | | | |
| Enrollment | 45.70 (2.50) | 46.46 (2.48) | - | - | 0.76 (-2.15, 3.67) | 0.608 |  |
| 1 | 44.95 (2.51) | 44.99 (2.49) | -0.75 (-2.91, 1.41) | -1.46 (-3.66, 0.73) | -0.72 (-3.79, 2.36) | 0.649 |  |
| 2 | 46.15 (2.51) | 44.95 (2.49) | 0.45 (-1.72, 2.62) | -1.50 (-3.67, 0.66) | -1.95 (-5.02, 1.11) | 0.212 |  |
| 3 | 49.78 (2.51) | 45.83 (2.48) | 4.08 (1.94, 6.23) | -0.63 (-2.77, 1.52) | -4.71 (-7.75, -1.68) | 0.002 |  |
| 4 | 47.23 (2.51) | 43.52 (2.49) | 1.53 (-0.61, 3.67) | -2.93 (-5.10, -0.76) | -4.47 (-7.51, -1.42) | 0.004 |  |
| 5 | 49.50 (2.50) | 46.35 (2.48) | 3.80 (1.68, 5.92) | -0.11 (-2.24, 2.02) | -3.91 (-6.92, -0.91) | 0.011 |  |
| HVLT Delayed Recall, estimated mean (SD) 0.007 | | | | | | | |
| Enrollment | 44.83 (2.51) | 45.94 (2.49) | - | - | 1.11 (-1.91, 4.14) | 0.470 |  |
| 1 | 45.23 (2.52) | 44.54 (2.50) | 0.40 (-1.95, 2.74) | -1.40 (-3.80, 0.99) | -1.80 (-5.15, 1.55) | 0.292 |  |
| 2 | 47.35 (2.52) | 45.37 (2.49) | 2.52 (0.15, 4.88) | -0.57 (-2.93, 1.79) | -3.09 (-6.43, 0.25) | 0.070 |  |
| 3 | 48.47 (2.52) | 44.84 (2.49) | 3.64 (1.30, 5.98) | -1.11 (-3.44, 1.23) | -4.75 (-8.06, -1.44) | 0.005 |  |
| 4 | 47.27 (2.51) | 42.64 (2.50) | 2.44 (0.11, 4.77) | -3.30 (-5.66, -0.94) | -5.74 (-9.05, -2.42) | 0.001 |  |
| 5 | 50.20 (2.51) | 46.65 (2.49) | 5.37 (3.06, 7.68) | 0.71 (-1.61, 3.03) | -4.66 (-7.94, -1.39) | 0.005 |  |

Abbreviations: FIB-4: Fibrosis-4; LNST: Letter Number Sequencing Test; SDMT: Symbol Digit Modalities Test; SFT: Semantic Fluency Test; HVLT: Hopkins Verbal Learning Test.

P^a^：P for Difference between subgroups; P^b^:P for FIB-4 subgroup*time

**Supplementary Table 4.** Longitudinal changes of cognitive-related scale scores in different FIB-4 subgroups in patients≤ 60 years old during 5 years

| **Clinical scales** | Low FIB-4 subgroup | High FIB-4 subgroup | Difference in Low FIB-4 subgroup(95% CI)) | Difference in high FIB-4 subgroup(95% CI) | Difference between subgroups(95% CI) | P^a^ | P^b^ |
| --- | --- | --- | --- | --- | --- | --- | --- |
| LSNT, estimated mean (SD) 0.272 | | | | | | | |
| Enrollment | 11.72 (0.60) | 11.38 (0.70) | - | - | -0.34 (-1.26, 0.58) | 0.466 |  |
| 1 | 11.27 (0.61) | 11.71 (0.72) | -0.45 (-0.94, 0.04) | 0.33 (-0.63, 1.30) | 0.78 (-0.30, 1.87) | 0.156 |  |
| 2 | 11.45 (0.61) | 11.83 (0.71) | -0.27 (-0.76, 0.21) | 0.45 (-0.47, 1.36) | 0.72 (-0.32, 1.76) | 0.174 |  |
| 3 | 11.43 (0.61) | 10.92 (0.70) | -0.29 (-0.77, 0.19) | -0.46 (-1.37, 0.44) | -0.17 (-1.20, 0.86) | 0.744 |  |
| 4 | 11.54 (0.61) | 11.11 (0.70) | -0.18 (-0.66, 0.30) | -0.27 (-1.18, 0.64) | -0.09 (-1.12, 0.94) | 0.865 |  |
| 5 | 11.28 (0.60) | 10.84 (0.70) | -0.45 (-0.92, 0.03) | -0.54 (-1.44, 0.37) | -0.09 (-1.12, 0.93) | 0.861 |  |
| SDMT, estimated mean (SD) 0.849 | | | | | | | |
| Enrollment | 47.73 (1.96) | 47.46 (2.29) | - | - | -0.27 (-3.30, 2.75) | 0.860 |  |
| 1 | 47.64 (1.97) | 47.22 (2.36) | -0.10 (-1.73, 1.54) | -0.24 (-3.49, 3.01) | -0.14 (-3.78, 3.49) | 0.938 |  |
| 2 | 47.56 (1.97) | 46.76 (2.30) | -0.17 (-1.81, 1.46) | -0.70 (-3.78, 2.38) | -0.53 (-4.02, 2.96) | 0.768 |  |
| 3 | 47.09 (1.97) | 48.31 (2.29) | -0.65 (-2.27, 0.98) | 0.85 (-2.20, 3.89) | 1.49 (-1.96, 4.94) | 0.397 |  |
| 4 | 47.56 (1.97) | 46.92 (2.29) | -0.18 (-1.79, 1.44) | -0.54 (-3.58, 2.51) | -0.36 (-3.81, 3.09) | 0.837 |  |
| 5 | 47.83 (1.96) | 48.46 (2.29) | 0.10 (-1.51, 1.70) | 1.00 (-2.05, 4.05) | 0.90 (-2.54, 4.35) | 0.607 |  |
| SFT, estimated mean (SD) 0.235 | | | | | | | |
| Enrollment | 53.46 (2.47) | 53.92 (2.87) | - | - | 0.46 (-3.33, 4.26) | 0.811 |  |
| 1 | 54.64 (2.48) | 51.17 (2.95) | 1.18 (-0.88, 3.25) | -2.74 (-6.86, 1.37) | -3.93 (-8.53, 0.68) | 0.095 |  |
| 2 | 54.49 (2.48) | 52.76 (2.88) | 1.03 (-1.04, 3.11) | -1.16 (-5.07, 2.74) | -2.20 (-6.62, 2.23) | 0.330 |  |
| 3 | 56.18 (2.48) | 51.84 (2.87) | 2.72 (0.67, 4.78); p=0.010 | -2.08 (-5.94, 1.78); p=0.292 | -4.80 (-9.17, -0.42) | 0.032 |  |
| 4 | 54.48 (2.48) | 52.77 (2.87) | 1.02 (-1.03, 3.07) | -1.15 (-5.02, 2.71) | -2.17 (-6.55, 2.20) | 0.330 |  |
| 5 | 57.10 (2.47) | 52.92 (2.87) | 3.65 (1.62, 5.68) | -1.00 (-4.86, 2.86) | -4.65 (-9.01, -0.29) | 0.037 |  |
| HVLT Total Recal, estimated mean (SD) 0.063 | | | | | | | |
| Enrollment | 46.19 (2.61) | 45.53 (3.03) | - | - | -0.65 (-4.67, 3.36) | 0.750 |  |
| 1 | 45.19 (2.62) | 46.26 (3.12) | -1.00 (-3.17, 1.18) | 0.73 (-3.62, 5.07) | 1.72 (-3.13, 6.58) | 0.487 |  |
| 2 | 46.22 (2.62) | 43.54 (3.04) | 0.03 (-2.16, 2.22) | -1.99 (-6.11, 2.13) | -2.02 (-6.69, 2.65) | 0.396 |  |
| 3 | 49.92 (2.62) | 45.42 (3.03) | 3.73 (1.56, 5.90) | -0.12 (-4.19, 3.96) | -3.85 (-8.46, 0.77) | 0.102 |  |
| 4 | 47.60 (2.62) | 42.11 (3.03) | 1.41 (-0.75, 3.58) | -3.42 (-7.50, 0.65) | -4.84 (-9.45, -0.22) | 0.040 |  |
| 5 | 49.77 (2.61) | 45.53 (3.03) | 3.59 (1.44, 5.73) | 0.00 (-4.07, 4.07) | -3.59 (-8.19, 1.02) | 0.127 |  |
| HVLT Delayed Recall, estimated mean (SD) 0.965 | | | | | | | |
| Enrollment | 44.80 (2.53) | 43.87 (2.97) | - | - | -0.93 (-5.01, 3.14) | 0.653 |  |
| 1 | 44.89 (2.54) | 44.92 (3.07) | 0.09 (-2.22, 2.40) | 1.05 (-3.54, 5.65) | 0.96 (-4.18, 6.10) | 0.714 |  |
| 2 | 46.38 (2.54) | 44.00 (2.99) | 1.58 (-0.74, 3.89) | 0.13 (-4.23, 4.49) | -1.45 (-6.38, 3.49) | 0.566 |  |
| 3 | 46.84 (2.54) | 45.64 (2.97) | 2.03 (-0.26, 4.33) | 1.77 (-2.54, 6.08) | -0.27 (-5.15, 4.62) | 0.915 |  |
| 4 | 45.94 (2.54) | 44.10 (2.97) | 1.13 (-1.16, 3.42) | 0.23 (-4.08, 4.54) | -0.90 (-5.78, 3.98) | 0.717 |  |
| 5 | 48.54 (2.53) | 47.22 (2.97) | 3.73 (1.47, 6.00) | 3.35 (-0.97, 7.66) | -0.39 (-5.26, 4.48) | 0.876 |  |

Abbreviations: FIB-4: Fibrosis-4; LNST: Letter Number Sequencing Test; SDMT: Symbol Digit Modalities Test; SFT: Semantic Fluency Test; HVLT: Hopkins Verbal Learning Test.

P^a^：P for Difference between subgroups; P^b^:P for FIB-4 subgroup*time

**Supplementary** **Table 5.** Longitudinal changes of cognitive-related scale scores in different FIB-4 subgroups in patients> 60 years old during 5 years

| **Clinical scales** | Low FIB-4 subgroup | High FIB-4 subgroup | Difference in Low FIB-4 subgroup(95% CI)) | Difference in high FIB-4 subgroup(95% CI) | Difference between subgroups(95% CI) | P^a^ | P^b^ |
| --- | --- | --- | --- | --- | --- | --- | --- |
| LSNT, estimated mean (SD) 0.084 | | | | | | | |
| Enrollment | 10.46 (0.52) | 10.80 (0.49) | - | - | 0.35 (-0.29, 0.98) | 0.289 |  |
| 1 | 10.66 (0.52) | 10.85 (0.49) | 0.20 (-0.36, 0.76) | 0.05 (-0.38, 0.47) | -0.15 (-0.86, 0.55) | 0.668 |  |
| 2 | 10.72 (0.52) | 10.51 (0.49) | 0.26 (-0.31, 0.82) | -0.30 (-0.72, 0.13) | -0.56 (-1.26, 0.15) | 0.124 |  |
| 3 | 10.39 (0.52) | 10.23 (0.49) | -0.07 (-0.63, 0.48) | -0.57 (-0.99, -0.15) | -0.50 (-1.20, 0.20) | 0.161 |  |
| 4 | 10.55 (0.52) | 10.11 (0.49) | 0.09 (-0.46, 0.65) | -0.69 (-1.12, -0.27) | -0.78 (-1.48, -0.09) | 0.028 |  |
| 5 | 10.26 (0.52) | 9.70 (0.49) | -0.20 (-0.75, 0.36) | -1.10 (-1.52, -0.68) | -0.91 (-1.60, -0.21) | 0.010 |  |
| SDMT, estimated mean (SD) 0.139 | | | | | | | |
| Enrollment | 41.12 (2.31) | 41.19 (2.20) | - | - | 0.07 (-2.59, 2.73) | 0.958 |  |
| 1 | 41.57 (2.32) | 40.58 (2.20) | 0.45 (-1.73, 2.62) | -0.62 (-2.26, 1.02) | -1.06 (-3.79, 1.66) | 0.445 |  |
| 2 | 40.04 (2.32) | 39.08 (2.20) | -1.08 (-3.25, 1.08) | -2.11 (-3.75, -0.47) | -1.03 (-3.75, 1.69) | 0.458 |  |
| 3 | 39.94 (2.31) | 38.73 (2.20) | -1.18 (-3.31, 0.95) | -2.46 (-4.09, -0.83) | -1.28 (-3.96, 1.40) | 0.350 |  |
| 4 | 38.83 (2.31) | 36.94 (2.20) | -2.29 (-4.42, -0.16) | -4.25 (-5.89, -2.62) | -1.96 (-4.65, 0.72) | 0.152 |  |
| 5 | 39.80 (2.31) | 36.18 (2.20) | -1.32 (-3.44, 0.80) | -5.01 (-6.62, -3.40) | -3.69 (-6.35, -1.03) | 0.007 |  |
| SFT, estimated mean (SD) 0.098 | | | | | | | |
| Enrollment | 47.93 (2.41) | 48.85 (2.30) | - | - | 0.93 (-1.96, 3.81) | 0.530 |  |
| 1 | 48.27 (2.43) | 49.46 (2.31) | 0.34 (-2.23, 2.92) | 0.61 (-1.32, 2.54) | 0.26 (-2.96, 3.48) | 0.873 |  |
| 2 | 48.83 (2.42) | 48.13 (2.31) | 0.90 (-1.65, 3.45) | -0.72 (-2.65, 1.21) | -1.62 (-4.82, 1.58) | 0.321 |  |
| 3 | 49.68 (2.41) | 48.79 (2.30) | 1.76 (-0.75, 4.26) | -0.06 (-1.97, 1.85) | -1.82 (-4.97, 1.34) | 0.259 |  |
| 4 | 51.07 (2.41) | 47.89 (2.30) | 3.15 (0.64, 5.65) | -0.96 (-2.89, 0.96) | -4.11 (-7.27, -0.95) | 0.011 |  |
| 5 | 48.89 (2.41) | 47.94 (2.30) | 0.96 (-1.53, 3.46) | -0.91 (-2.80, 0.99) | -1.87 (-5.00, 1.26) | 0.242 |  |
| HVLT Total Recal, estimated mean (SD) 0.015 | | | | | | | |
| Enrollment | 47.50 (2.51) | 48.09 (2.37) | - | - | 0.59 (-2.46, 3.64) | 0.704 |  |
| 1 | 46.08 (2.53) | 46.18 (2.38) | -1.42 (-4.15, 1.30) | -1.91 (-3.95, 0.13) | -0.49 (-3.89, 2.92) | 0.780 |  |
| 2 | 48.37 (2.52) | 46.22 (2.38) | 0.87 (-1.83, 3.56) | -1.86 (-3.91, 0.18) | -2.73 (-6.11, 0.65) | 0.113 |  |
| 3 | 51.58 (2.51) | 47.80 (2.37) | 4.08 (1.43, 6.73) | -0.29 (-2.31, 1.73) | -4.38 (-7.71, -1.04) | 0.010 |  |
| 4 | 50.23 (2.51) | 45.91 (2.37) | 2.73 (0.08, 5.38) | -2.18 (-4.21, -0.14) | -4.90 (-8.24, -1.56) | 0.004 |  |
| 5 | 50.68 (2.51) | 47.53 (2.37) | 3.18 (0.54, 5.82) | -0.56 (-2.56, 1.45) | -3.74 (-7.05, -0.42) | 0.027 |  |
| HVLT Delayed Recall, estimated mean (SD) 0.006 | | | | | | | |
| Enrollment | 45.69 (2.62) | 47.39 (2.48) | - | - | 1.70 (-1.54, 4.95) | 0.303 |  |
| 1 | 45.87 (2.64) | 45.96 (2.49) | 0.18 (-2.75, 3.12) | -1.43 (-3.64, 0.78) | -1.61 (-5.29, 2.06) | 0.390 |  |
| 2 | 48.07 (2.64) | 46.78 (2.49) | 2.38 (-0.54, 5.30) | -0.61 (-2.82, 1.60) | -2.99 (-6.65, 0.67) | 0.109 |  |
| 3 | 49.72 (2.63) | 46.51 (2.48) | 4.03 (1.16, 6.90) | -0.88 (-3.07, 1.31) | -4.91 (-8.51, -1.30) | 0.008 |  |
| 4 | 49.92 (2.63) | 45.02 (2.49) | 4.23 (1.36, 7.10) | -2.37 (-4.57, -0.16) | -6.60 (-10.22, -2.98) | <0.001 |  |
| 5 | 50.37 (2.62) | 47.89 (2.48) | 4.68 (1.82, 7.53) | 0.49 (-1.67, 2.66) | -4.18 (-7.77, -0.60) | 0.022 |  |

Abbreviations: FIB-4: Fibrosis-4; LNST: Letter Number Sequencing Test; SDMT: Symbol Digit Modalities Test; SFT: Semantic Fluency Test; HVLT: Hopkins Verbal Learning Test.

P^a^：P for Difference between subgroups; P^b^:P for FIB-4 subgroup*time
